# Macrophage GIT1 Contributes to Bone Regeneration by Regulating Inflammatory Responses in an ERK/NRF2‐Dependent Way

**DOI:** 10.1002/jbmr.4099

**Published:** 2020-07-09

**Authors:** Shu‐Jie Zhao, Hao Liu, Jian Chen, Ding‐Fei Qian, Fan‐Qi Kong, Jian Jie, Guo‐Yong Yin, Qing‐Qing Li, Jin Fan

**Affiliations:** ^1^ Department of Orthopedics The First Affiliated Hospital of Nanjing Medical University Nanjing China; ^2^ Department of Orthopedics Pukou Branch of Jiangsu Province Hospital (Nanjing Pukou Central Hospital) Nanjing China

**Keywords:** GENETIC ANIMAL MODELS, ANIMAL MODELS, STROMAL/STEM CELLS, CELLS OF BONE, INJURY/FRACTURE HEALING, ORTHOPEDICS

## Abstract

Despite the best treatment, approximately 10% of fractures still face undesirable repair. Recently, many studies have focused on the importance of macrophages in bone repair; however, the cellular mechanisms by which they work are not yet fully understood. In this study, we explored the functions of macrophage G‐protein‐coupled receptor interacting protein 1 (GIT1) in healing a tibial monocortical defect model. Using GIT1^flox/flox^ Lyz2‐Cre (GIT1 CKO) mice, we observed that a GIT1 deficiency in the macrophages led to an exacerbation of interleukin 1β (IL1β) production, more M1‐like macrophage infiltration, and impaired intramembranous ossification in vivo. The results of in vitro assays further indicated that the macrophage GIT1 plays a critical role in several cellular processes in response to lipopolysaccharide (LPS), such as anti‐oxidation, IL1β production alleviation, and glycolysis control. Although GIT1 has been recognized as a scaffold protein, our data clarified that GIT1‐mediated extracellular‐signal‐regulated kinase (ERK) phosphorylation could activate nuclear factor (erythroid‐derived 2)‐like 2 (NRF2) in macrophages after LPS treatment. Moreover, we demonstrated that macrophage GIT1‐activated ERK/NRF2 negatively regulates the 6‐phosphofructo‐2‐kinase/fructose‐2, 6‐biphosphatase 3 (PFKFB3), facilitating the decrease of glycolysis. Our findings uncovered a previously unrecognized role of GIT1 in regulating ERK/NRF2 in macrophages to control the inflammatory response, suggesting that macrophage GIT1 could be a potential target to improve bone regeneration. © 2020 The Authors. *Journal of Bone and Mineral Research* published by American Society for Bone and Mineral Research..

## Introduction

Skeletal fracture is the most common issue treated by orthopedic surgeons, leading to a heavy annual national economic burden.^(^ ^1^ ^)^ During normal fracture repair, bones have a strong capacity for self‐repair.^(^ ^2^ ^)^ Intramembranous ossification (IO) and endochondral ossification (EO) are the two major paths of bone regeneration.^(^ ^3^, ^4^ ^)^ In IO, the direct formation of a bone callus without a cartilaginous intermediary is involved in the direct osteogenic differentiation of mesenchymal stem cells (MSCs).^(^ ^3^, ^4^ ^)^ During EO, an intermediary cartilaginous callus is gradually replaced by bone.^(^ ^3^, ^4^ ^)^ However, delayed or non‐union fracture healing still represents a critical clinical challenge, as up to 10% of fractures result in undesirable outcomes.^(^ ^5^ ^)^ Thus, a better understanding of the mechanisms underlying fracture repair is critical to improve clinical outcomes.

Optimal fracture healing requires a transient acute inflammatory reaction, followed by resolution of the inflammatory reaction and then the tissue regeneration phase.^(^ ^6^, ^7^ ^)^ Accumulating evidence has shown that macrophages are the main players during the above activities.^(^ ^8^ ^)^ Macrophages have the capacity to exhibit varied subtypes in response to environmental stimuli.^(^ ^9^ ^)^ During the first 3 days after a bone fracture, the inflammatory cue that follows polarizes macrophages toward an M1‐like phenotype (pro‐inflammatory).^(^ ^10^, ^11^ ^)^ These M1‐like macrophages are characteristic of enhanced glycolysis, which is important for the production of pro‐inflammatory cytokines.^(^ ^12^, ^13^, ^14^ ^)^ Then, the M1‐like macrophages are gradually replaced by an M2‐like phenotype (anti‐inflammatory). By day 7, the M2‐like macrophages become the dominant population and favor tissue repair at later stages.^(^ ^10^, ^11^ ^)^ Dysregulation of the above process can lead to imbalanced inflammation and impaired bone regeneration. However, the relative details and exact mechanisms by which macrophages contribute to bone regeneration have long been obscure.

Recently, evidence has emerged showing the cytoprotective function of nuclear factor (erythroid‐derived 2)‐like 2 (NRF2) in the inflammatory network. It plays a crucial role in allergies, autoimmune diseases, cancer, and fracture repair.^(^ ^15^, ^16^, ^17^ ^)^ Moreover, it is reported to be expressed in macrophages and plays a key role in regulating oxidative stress and inhibiting interleukin‐1β (IL1β) production.^(^ ^18^, ^19^, ^20^ ^)^ In addition to the well‐known regulation of NRF2 by kelch‐like ECH associated protein 1 (Keap1), the expression and activity of NRF2 can be modulated in various ways, such as post‐transcriptionally regulated by micro‐RNAs (miR‐28, 34, 144), post‐translationally phosphorylated by a variety of proteins (extracellular‐signal‐regulated kinase [ERK], phosphoenolpyruvate carboxykinase, p38), and acetylated by p300/CBP.^(^ ^16^, ^21^ ^)^ The importance of NRF2 regulation in macrophages during fracture repair has yet to be examined; thus, the fine mechanisms of this process require further study.

G‐protein‐coupled receptor interacting protein 1 (GIT1) is a member of the GIT family that contains a variety of functional domains and serves as a scaffold protein.^(^ ^22^, ^23^ ^)^ Several previous studies have established that GIT1 plays a key role in the mitogen‐activated protein kinase (MAPK) pathway, which is required for the activation of MEK1‐ERK1/2.^(^ ^24^, ^25^, ^26^ ^)^ Furthermore, we previously demonstrated the regulating role of GIT1 in EO post‐fracture in a mouse femur fracture model, showing that the depletion of GIT1 impairs EO via lessening the callus vascularity, decreasing the chondrocyte proliferation, and reducing the osteoclast (OC) number.^(^ ^27^, ^28^ ^)^ However, the specific function of the macrophage GIT1 during IO and whether GIT1‐mediated regulation of NRF2 exists in macrophages remain unclear.

In this study, we observe that GIT1 CKO mice display delayed IO compared with GIT1^flox/flox^ (GIT1^fl/fl^) mice in a tibial monocortical defect model. Notably, an increased portion of M1‐like macrophages, upregulated reactive oxygen species (ROS) production, and enhanced glycolysis are observed in GIT1‐deficient bone marrow–derived macrophages (BMDMs) in response to lipopolysaccharide (LPS). The abnormally upregulated secretion of IL1β from GIT1‐deficient LPS‐activated BMDMs could lead to decreased osteoinductive effect to bone marrow stromal cells (BMSCs). Mechanismly, our study revealed that GIT1 facilitated to phosphorylate ERK in LPS‐activated macrophages, which allows NRF2 to stabilize and translocate into the nucleus for promoting antioxidative gene transcription and inhibiting expression of IL1β and 6‐phosphofructo‐2‐kinase/fructose‐2, 6‐biphosphatase 3 (PFKFB3). Our findings indicated a previously unrecognized role of GIT1 in regulating ERK/NRF2 in LPS‐activated macrophages to control the inflammatory response.

## Materials and Methods

### Cell culture and reagents

The murine macrophage cell line RAW264.7 was obtained from the Cell Bank of the Chinese Academy of Sciences (Shanghai, China) and cultured as previously described.^(^ ^29^ ^)^ Furthermore, the isolation and culture of primary BMDMs and BMSCs were performed as previously described.^(^ ^29^ ^)^ The polarization of BMDMs and RAW264.7 cells toward the M1‐like phenotype was performed by adding 100 ng/mL LPS (L2880, Sigma‐Aldrich, St. Louis, MO, USA) to the macrophage culture media. Mouse‐derived bone marrow cells were treated with 50 ng/mL RANKL (462‐TEC, R&D Systems, Minneapolis, MN, USA) and 20 ng/mL M‐CSF (416‐ML, R&D Systems) for inducing osteoclast formation. Primary osteoblasts were obtained from calvarial bones of newborn mice as previously described.^(^ ^30^ ^)^ Briefly, the calvariae were digested with collagenase (V900892, Sigma‐Aldrich) and Dispase II (4942078001, Roche, Basel, Switzerland) and maintained in α‐MEM (41061037, GIBCO, Grand Island, NY, USA) with 10% FBS.^(^ ^30^ ^)^ Chondrocytes were obtained from the knees of 5‐day‐old mice. In brief, after washing with PBS, the cartilage was cut into pieces. Next, cartilage chips were sequential incubated with trypsin–EDTA solution and 3 mg/mL Collagenase D (11088882001, Roche). The digested cartilage chips and released cells were washed twice and plated in a 10‐cm dish. The antibodies for Western blotting used in our study included anti‐β‐actin (AB0011, Abways, Shanghai, China); anti‐Histone H3 (3638, CST, Danvers, MA, USA); anti‐GIT1 (S39B‐8, NOVUS, Littleton, CO, USA); anti‐NRF2 (12721 T, CST); anti‐NAD(P)H:quinone oxidoreductase 1 (NQO1) (3187, CST); anti‐heme oxygenase 1 (HO1) (10701‐1‐AP, Proteintech, Wuhan, China); anti‐ERK (4695 T, CST); anti‐p‐ERK (4370 T, CST); anti‐IL1β (31202, CST); and anti‐PFKFB3 (13123, CST). The secondary antibodies for the Western blotting were purchased from Jackson ImmunoResearch (West Grove, PA, USA). The antibodies used for the flow cytometry were as follows: F4/80‐PE (565410, BD, Franklin Lakes, NJ, USA); iNOS‐FITC (610330, BD); CD206‐APC (17–2061‐82, Thermo Fisher Scientific, Waltham, MA, USA); and CD11b‐FITC (557396, BD). The antibodies for the immunofluorescence (IF) included anti‐iNOS (ab15323, Abcam, Cambridge, UK); anti‐CD206 (ab64693, Abcam); anti‐F4/80 (14–4801‐82, Thermo). The secondary antibodies used for the IF were donkey anti‐mouse Alexa Fluor 488 (ab150105, Abcam); goat anti‐rabbit Alexa Fluor 594 (ab150088, Abcam); and goat anti‐rabbit Alexa Fluor 647 (ab150083, Abcam). Further, the nuclei were stained using DAPI (D9542, Sigma‐Aldrich). Osteoprotegerin (OPG) (450‐14, Pepro‐Tech, Rocky Hill, NJ, USA) was used for osteoclast depletion. Moreover, clodronate liposomes and control liposomes (Encapsula NanoSciences, Brentwood, TN, USA) were acquired for monocyte/macrophage depletion. Recombinant Mouse IL1β protein (401‐ML) and neutralizing antibody against IL1β (MAB401) were both obtained from R&D. To inhibit ERK, we purchased SCH772984 (S7101, Selleckchem, Houston, TX, USA), and diethyl maleate (DEM) (D97703, Sigma‐Aldrich) was obtained for activating NRF2. N‐Acetyl‐l‐cysteine (NAC, A9165, Sigma‐Aldrich) was used as an ROS scavenger.

### Generation of GIT1 CKO mice

GIT1^fl/fl^ and Lyz2‐Cre mice were both acquired from Gem Pharmatech Co., Ltd. (Nanjing, China). For producing myeloid‐specific knockout mice, GIT1^fl/fl^ mice were hybridized with Lyz2‐Cre mice. In this study, mice with Lyz2‐specific deletion of GIT1 were defined as CKO mice, and the GIT1^fl/fl^ mice were defined as the controls. The genomic DNA isolated from the mouse tails was analyzed by PCR and primers are listed in Supplemental Table S1. Furthermore, Western blotting was used for verification at the protein level. Housing and all experimental animal procedures were approved by the Animal Committee at the First Affiliated Hospital of Nanjing Medical University.

### Tibial monocortical defect model

Skeletal mature GIT1^fl/fl^ and GIT1 CKO mice (C57BL/6 background, 8‐week‐old males) (*n* = 3) were used to performed tibial monocortical defect model as previously described.^(^ ^29^ ^)^ Under anesthetic conditions, a monocortical osseous defect (0.8 mm in diameter) was drilled on the anterior surface of the tibia crest using a round burr attached to a dental drill (NSK Ultimate XL; NSK/Nakanishi, Kanuma Tochigi, Japan). The muscle and skin layers were closed, and buprenorphine was administered as an analgesic.

### Clodronate liposome, OPG, and anti‐IL1β neutralizing antibody treatment during bone healing

GIT1^fl/fl^ and GIT1 CKO mice were administered OPG via intradefect injection at the time (day 0) of surgery plus subcutaneous injections every second day (ie, days 2, 4, and 6) postoperatively (Supplemental Fig. S1 *C*).^(^ ^31^ ^)^ OPG was delivered at a final dose of 2 mg/kg per defect region. Saline was served as a control. The osteoclast depletion efficiency was analyzed using tartrate‐resistant acid phosphatase (TRAP) staining. For depleting the monocyte/macrophage, clodronate liposomes were used as described previously.^(^ ^32^ ^)^ GIT1^fl/fl^ and GIT1 CKO mice were first administered clodronate liposomes or control liposomes intravenously 2 days before tibial surgery. Then additional clodronate liposomes or control liposomes were intravenously injected every 2 days (ie, days 0, 2, 4, and 6) postoperatively (Supplemental Fig. S1 *C*). The monocyte/macrophage depletion efficiency was assessed by the percentage of CD11b^+^ cells in peripheral blood samples using flow cytometry assay. In IL1β neutralization assay, GIT1^fl/fl^ and CKO mice received i.p. injections of 100 μg anti‐IL1β neutralizing antibody 8 hours before tibial surgery and every 3 days postoperatively.^(^ ^33^, ^34^ ^)^

### Micro‐CT imaging

The tibias were harvested and then scanned using a micro‐CT analysis system (SkyScan 1176, Bruker microCT, Kontich, Belgium) as previously reported.^(^ ^29^ ^)^ A three‐dimensional (3D) histomorphometric analysis including bone volume/tissue volume (BV/TV); trabecular number (Tb.N); trabecular separation (Tb.Sp), and trabecular thickness (Tb.Th) was performed using CT‐Analyzer (CTAn, Bruker).

### Tartrate‐resistant acid phosphatase (TRAP) staining

The fixed cells and tissues were subjected to TRAP staining using the Acid Phosphatase, Leukocyte (TRAP) Kit (387A; Sigma‐Aldrich) as previously described.^(^ ^28^, ^35^ ^)^ TRAP‐positive cells with more than three nuclei were counted as mature osteoclasts and observed by an inverted microscopy (Olympus Corporation, Tokyo, Japan).

### Hematoxylin and eosin (H&E) staining and IF assay

The fixed samples were decalcified for 28 days, followed by embedding for sectioning and staining with H&E. The tissue IF staining was performed using a previously reported protocol.^(^ ^29^ ^)^ The primary antibodies against F4/80 (1:100), iNOS (1:100), and CD206 (1:100) were used. Subsequently, the sections were incubated with secondary fluorescent antibodies (donkey anti‐mouse Alexa Fluor 488 [1:400], goat anti‐rabbit Alexa Fluor 594 [1:400], and goat anti‐rabbit Alexa Fluor 647 [1:300]). The sections were further stained with DAPI, and a confocal microscope (Zeiss LSM710, Heidenheim, Germany) was used to obtain images. To quantify the positive cells in the bone defect region, three randomly selected fields in each sample were photographed and the numbers were counted using ImageJ.

### Alizarin red staining and alkaline phosphatase enzyme assay

To detect calcification during osteogenic differentiation, the fixed BMSCs were stained with 2% alizarin red for 20 minutes as previously performed.^(^ ^29^ ^)^ The images were obtained with a Nikon camera (D750, Nikon Corporation, Tokyo, Japan). To quantify the mineralization, the stained cells were dissolved with cetylpyridinium chloride, and the alizarin red absorbance was assayed at 562 nm.

For evaluating the deposited mineral, the alkaline phosphatase (ALP) activity was analyzed with a BCIP/NBT alkaline phosphatase color development kit (C3206, Beyotime, Shanghai, China) in accordance with the manufacturer's instructions. For the colorimetric measurement of the ALP activity, the absorbance at 405 nm was measured using a spectrophotometer. Moreover, pixel quantification of pictures was performed using ImageJ.^(^ ^36^ ^)^

### Elisa

The tumor necrosis factor alpha (TNFα) and IL1β levels from cell supernatant were measured using their corresponding specific ELISA kits (70‐EK282HS‐96 and 70‐EK201BHS‐96, MultiSciences, Hangzhou, China) in accordance with the manufacturer's protocol. The ELISA kits were also used to test the level of TNFα and IL1β from tissue. Briefly, at indicated time points post‐injury, we collected the bone tissue and partially remodeled matrix within 1 mm from both ends of the defect area (along the long shaft). Each sample was then incubated in 1 mL of tissue protein extraction reagent (78510, Thermo Fisher) plus EDTA‐free complete protease inhibitor cocktail tablets (11836170001, Roche) and homogenized with a tissue homogenizer. Tissue lysates were incubated 1 hour at 37 °C and centrifuged at 12,000 rpm for 5 minutes. The supernatants were transferred to a fresh tube and stored at −80 °C until ELISA assessment.

### Flow cytometry

To detect the portion of M1‐like macrophages in vitro, single‐cell suspensions of BMDMs were incubated with F4/80‐PE and iNOS‐FITC according to the manufacturer's instructions. To test the efficiency of monocyte/macrophage depletion, the samples were incubated with CD11b‐FITC. All the labeled cells were determined using flow cytometry (FACSVerse 8, BD), and the FlowJo software (Version 10.6.1, TreeStar, Ashland, OR, USA) was carried out to analyze the data.

### Cell counting kit (CCK)‐8 assay

CCK‐8 assay (Dojindo Molecular Technologies, Kumamoto, Japan) was carried out following the vendor's instructions. In brief, BMSCs (3 × 10^3^) were cultured in 96‐well plates with conditioned media (CM) from LPS‐activated GIT1^fl/fl^ BMDMs (GIT1^fl/fl^ CM) or CM from LPS‐activated GIT1‐depleted BMDMs (CKO CM). The optical absorbance at 450 nm was detected using a plate reader (Thermo Fisher) at 0, 24, 48, 72, and 96 h.

### ROS evaluation

The ROS levels were measured using the ROS Assay Kit (S0033, Beyotime) through the oxidative conversion of cell‐permeable 2′,7′‐dichlorodihydrofluorescein diacetate to fluorescent dichlorofluorescein. Briefly, the cells were collected and incubated with DCFH‐DA according to the manufacturer's instructions. Then, the cells were washed three times with serum‐free medium and transferred to polypropylene FACS tubes. The fluorescent signal intensity of DCF was read at 488 nm and 525 nm for excitation and emission, respectively.

### Analysis of reduced/oxidized glutathione (GSH/GSSG) ratio

The ratio of GSH/GSSG was evaluated in BMDMs using the GSH and GSSG Assay Kit (S0053, Beyotime). All steps in the procedure were performed according to the manufacturer's instructions.

### Measurement of glycolysis

The glycolysis of BMDMs and RAW264.7 cells from different groups was quantified by the XF96 Metabolic Flux Analyzer (Seahorse Biosciences, Billerica, MA, USA) as previously described.^(^ ^29^, ^37^ ^)^ In brief, the extracellular acidification rate (ECAR) was determined by the sequential injection of glucose, oligomycin (Sigma‐Aldrich), and 2‐deoxyglucose (D8375; Sigma‐Aldrich).^(^ ^38^ ^)^ The instrument recorded 12 measurements for the ECAR, which was then measured via the XFe Wave software (Seahorse Biosciences). Additionally, the glycolysis and glycolytic capacity were calculated according to the manufacturer's protocol.

### RNA isolation and qPCR

Total RNA was extracted using the trizol reagent (Takara, Dalian, China), and the cDNA was amplified using the HiScript II Q RT SuperMix for qPCR (R122‐01, Vazyme, Nanjing, China) according to the manufacturer's instructions. The qPCR was performed by a real‐time 7500 PCR system (Applied Biosystems, Inc., Carlsbad, CA, USA) using AceQ qPCR SYBR Green Master Mix (Q111‐02, Vazyme). All primer sequences are listed in Supplemental Table S2. The target genes were normalized to β‐actin expression, and the relative expression levels were analyzed using the 2^−△CT^ method.

### Western blotting

Total protein and nucleoprotein were extracted from cells as previously reported.^(^ ^39^ ^)^ Equal amounts of the proteins were separated via sodium dodecyl sulfate polyacrylamide gel electrophoresis and transferred to a polyvinylidene fluoride membrane. After blocked with 5% skimmed milk or 5% bovine serum albumin, the membrane was incubated overnight at 4°C with primary antibodies. The primary antibodies used were as follows: anti‐β‐actin (1:2000), anti‐Histone H3 (1:1000), anti‐GIT1 (1:1000), anti‐NRF2 (1:1000), anti‐NQO1 (1:1000), anti‐HO1 (1:1000), anti‐ERK (1:1000), anti‐p‐ERK (1:1000), anti‐IL1β (1:1000), and anti‐PFKFB3 (1:1000). Next, immunodetections were performed using the appropriate secondary antibodies (1:10,000), and the immunoreactive bands were visualized via the Odyssey Imaging System (LI‐COR, Lincoln, NE, USA). Quantification of band intensity was also performed by ImageJ.

### Short interfering RNA (siRNA) transfection

To start, BMDMs were seeded onto six‐well plates at 60% to 65% confluence and then transfected with NRF2‐targeted siRNA or a negative control using the Lipofectamine RNAiMAX Reagent (Thermo Fisher).^(^ ^19^ ^)^ All transfections were performed according to the manufacturer's protocols. The siRNA sequences are listed in Supplemental Table S2. The knockdown efficiency was detected by Western blotting.

### Plasmid construction and transfection

All plasmids (GIT1 and vector) were constructed from FulenGen Ltd., Co. (Guangzhou, China). For plasmid transfection, RAW264.7 cells were transfected using Lipofectamin3000 (Thermo Fisher) transfection reagents following the product manual. The overexpression efficacy of GIT1 was verified by qPCR and Western blotting 48 hours after plasmid transfection.

### RNA sequence (RNA‐seq)

The total RNA of LPS‐activated BMDMs derived from GIT1 CKO and GIT1^fl/fl^ mice (*n* = 3) was first extracted. Next, quality RNA samples were converted into cDNA libraries according to previously described methods.^(^ ^29^ ^)^ The purified fragments were enriched with 12 to 15 cycles of PCR to generate the cDNA libraries. Then, the libraries were sequenced using Illumina Hiseq X Ten according to the manufacturer's protocol. Additionally, the fragments per kilobase per million (FPKM) values of the genes were calculated, Pearson's correlation analysis was performed, and heat maps were created. The RNA‐seq results were uploaded to the Gene Expression Omnibus database with accession number GSE144739. In this study, differentially expressed genes (DEGs) were defined as fold changes >1.5 and *p* < 0.05, and Kyoto Encyclopedia of Genes and Genomes (KEGG) and Gene Ontology (GO) analyses were conducted to explore their biological significance.

### Chromatin immunoprecipitation (CHIP) assay

To start, integrative genomics viewer (IVG) was carried out to uncover the potential NRF2 binding sites on the regulatory regions of PFKFB3 based on CHIP‐sequence data (GSE 36030) from Gene Expression Omnibus database.^(^ ^40^ ^)^ After that, BMDMs were fixed using 1% (w/v) formaldehyde for 10 minutes at 37°C. Based on previously published methods,^(^ ^29^, ^37^ ^)^ the subsequent steps were performed using the Pierce Agarose CHIP Kit (26156, Thermo Fisher). The fixed samples underwent cross‐link and sonication processes. Next, DNA fragments were obtained and immunoprecipitated with corresponding antibodies (NRF2‐specific antibody or rabbit IgG) overnight at 4°C. Finally, the DNA was analyzed via qPCR using SYBR Green Master Mix and designed primers (Takara, Kusatsu, Japan). The primers used for the CHIP‐qPCR assay are listed in Supplemental Table S2.

### Bone marrow transplantation

As described previously,^(^ ^29^ ^)^ 8‐week‐old male CKO mice were lethally irradiated with 700 cGray from an X‐ray source (RS 2000 Pro, Radsource, Brentwood, TN, USA) before transplantation. Each lethally irradiated recipient mouse was injected with 5 × 10^6^ donor (GIT1^fl/fl^ or CKO mice) bone marrow cells via a tail vein injection (*n* = 3/per group). Both 7 days before and 14 days after transplantation, each recipient mouse was given water containing neomycin and polymyxin B. The recipient mice underwent tibial monocortical defect surgery 4 weeks after transplantation.

### Statistical analyses

In all cases, data were presented as box plots with median and interquartile range. GraphPad Prism 7 (GraphPad Software, La Jolla, CA, USA) was used to manipulate statistical analyses. Comparisons between the two groups were analyzed via an unpaired two‐tailed Student's *t* test and one‐way ANOVA analysis followed by the Tukey's post hoc test of variance for multiple comparisons. Further, comparisons between two groups in which are two different variables, two‐way ANOVA with Sidak's multiple comparison test were performed. Actual *p* value (up to 0.10) was indicated for each analysis.

## Results

### GIT1 depletion in macrophages resulted in a decreased IO

**Fig. 1.**
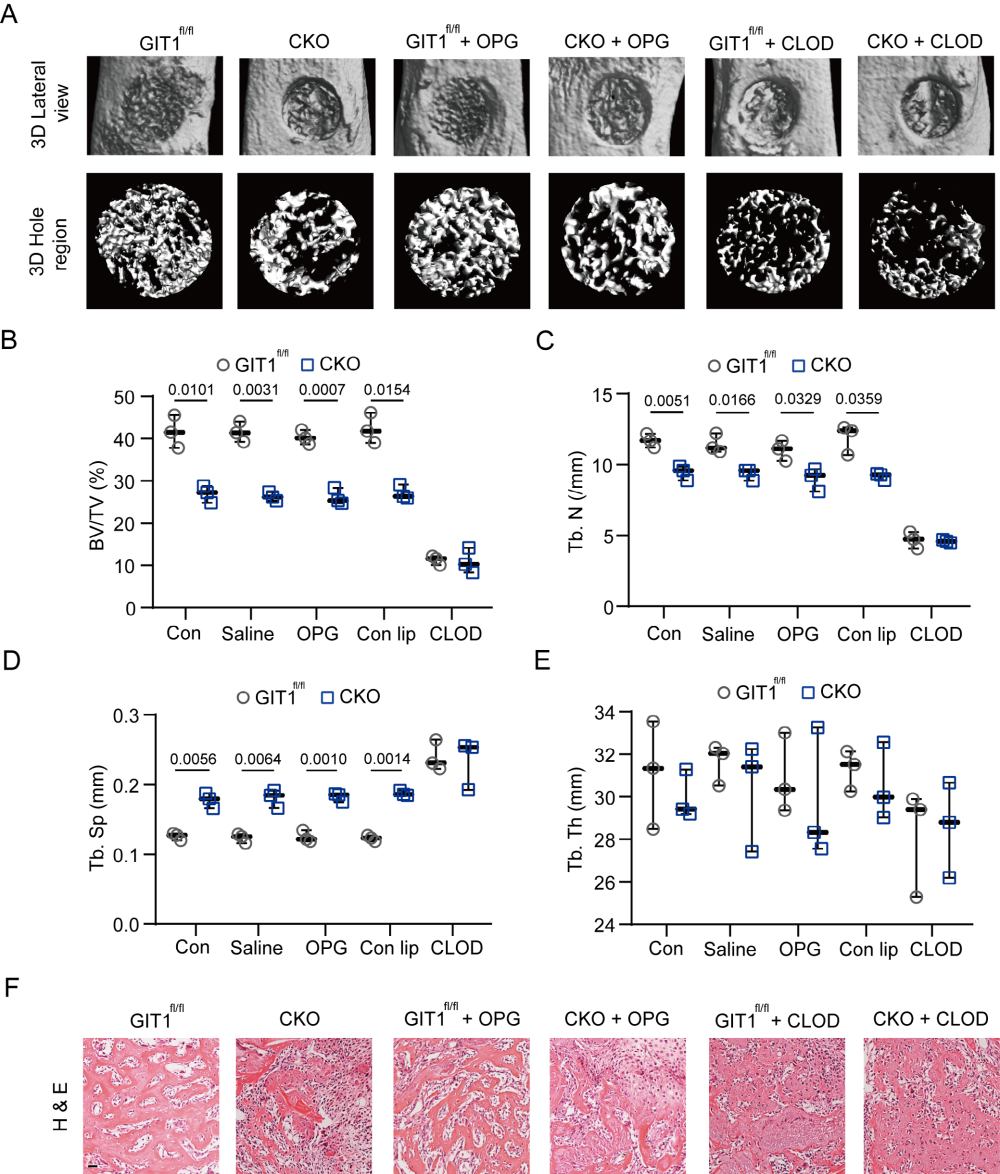
GIT1 CKO mice display impaired intramembranous bone formation of defects on day 7 post‐injury. (*A*) Representative images of micro‐CT reconstruction of injured tibias (top panel) and mineralized callus (lower panel) in the defect area of indicated groups. (*B–E*) BV/TV (%) (*B*), Tb.N (*C*), Tb.Sp (*D*), and Tb.Th (*E*) of the mineralized bone formed in the hole region were analyzed by micro‐CT (one‐way ANOVA with post hoc test). (*F*) Representative images of H&E staining in the hole region of indicated groups. Scale bar = 100 μm. BV = bone volume; TV = tissue volume; Tb.N = trabecular number; Tb.Sp = trabecular separation; Tb.Th = trabecular thickness; H&E = hematoxylin and eosin; OPG = osteoprotegerin; CLOD = clodronate liposomes.

To investigate the role of the macrophage GIT1 in IO, GIT1 CKO mice were first generated (Supplemental Fig. S1 *A*, *B*), and a tibial monocortical defect model was created. As shown in Fig. 1 *A*, {FIG1} the results of the 3D reconstruction of injured tibia showed less mineralized tissue in the GIT1 CKO mice than the controls on day 7 post‐injury. Furthermore, the BV/TV and Tb.N were decreased in the GIT1 CKO mice (Fig. 1*B*, *C*). In addition, compared with GIT1^fl/fl^ mice, the mineralized bone in the defects of the GIT1 CKO mice exhibited increased Tb.Sp, whereas the Tb.Th was not significantly changed (Fig. 1*D*, *E*). Histologically, in the GIT1 CKO mice, there was reduced organized bone regeneration and filling with more connective tissues, as revealed via H&E staining (Fig. 1*F*). Because GIT1 was deficient in both the macrophages and osteoclasts in the GIT1 CKO mice, osteoprotegerin (OPG) (osteoclast depletion) and clodronate liposomes (macrophage depletion) were used to determine the cell types contributing to the impaired IO. Additionally, the efficiency of the monocyte/macrophage and osteoclast depletion was confirmed (Supplemental Fig. S1 *C–F*). As shown in Fig. 1*A–F* and Supplemental S1 *G*, the 3D reconstruction images, morphometric parameter analysis results, and H&E staining consistently indicated that macrophages are essential for IO and decreased bone regeneration ability was mostly attributed to the macrophage GIT1 knockout but not to the osteoclast GIT1 depletion.

### GIT1 CKO mice displayed upregulated IL1β production and increased proportion of M1‐like macrophages in vivo and in vitro

**Fig. 2.**
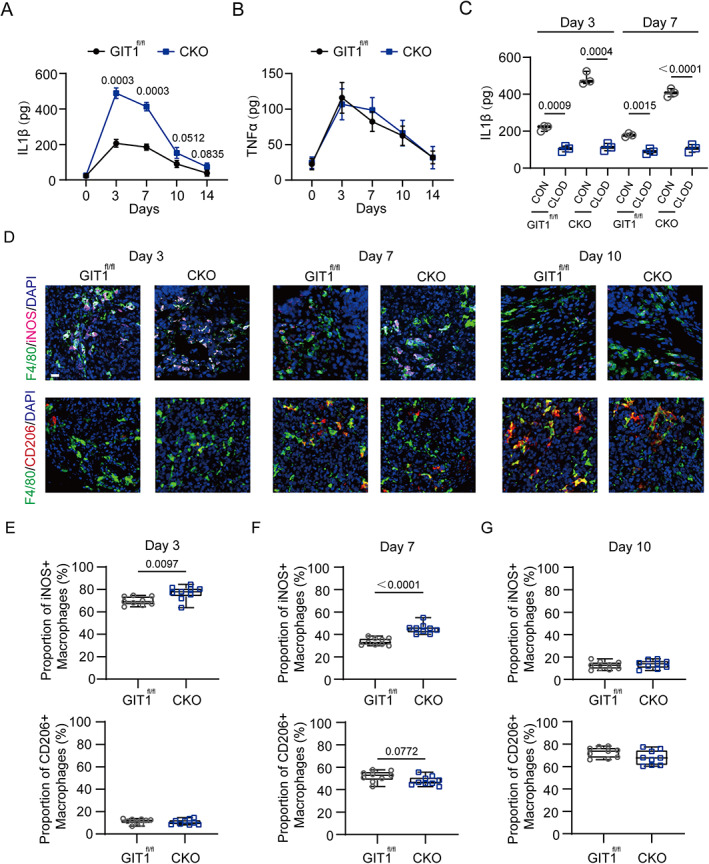
Enhanced IL1β production and higher proportion of M1‐like macrophages infiltrated in bone defects of GIT1 CKO mice on days 3 and 7 post‐injury. (*A*, *B*) Concentration of IL1β (*A*) and TNFα (*B*) present in the bone defect region at five time points (days 0, 3, 7, 10, and 14) after injury from GIT1^fl/fl^ and GIT1 CKO mice were measured by ELISA (one‐way ANOVA with post hoc test). (*C*) IL1β concentrations determined in GIT1^fl/fl^ and GIT1 CKO mice treated with clodronate liposomes (CLOD) or control liposomes (CON) (one‐way ANOVA with post hoc test). (*D*) In the tibial defect region, infiltrated M1‐like macrophages (top panel) were shown by IF staining with F4/80 (green) and iNOS (purple). M2‐like macrophages (lower panel) were present via IF staining with F4/80 (green) and CD206 (red) on days 3, 7, and 10 post‐injury. Nuclei were counterstained with DAPI (blue). Scale bar = 100 μm. (*E–G*) Statistical analysis of the proportion of infiltrated M1‐like (F4/80^+^ and iNOS^+^) and M2‐like (F4/80^+^ and CD206^+^) macrophages at three time points post‐injury (unpaired two‐tailed Student's *t* test).

It is well known that inflammation plays a critical role during fracture healing and that macrophages are the major players in the modulation of inflammatory reactions.^(^ ^6^, ^7^, ^8^, ^41^ ^)^ Therefore, we investigated whether macrophage GIT1 is involved in the regulation of inflammation. Based on a literature review^(^ ^6^, ^32^, ^42^, ^43^ ^)^ and enzyme‐linked immunosorbent assay (ELISA), we tested the expression patterns of representative pro‐inflammatory cytokines (IL1β and TNFα) present in the tibial defect microenvironment during the first 2 weeks post‐injury. Only IL1β was expressed abnormally on days 3 and 7 post‐injury in the GIT1 CKO mice (Fig. 2*A*, *B*). {FIG2} Furthermore, to determine the cell types secreting IL1β, we calculated the concentration of the cytokine in the bone defects of mice (both GIT1 CKO and GIT1^fl/fl^ groups) in which monocytes/macrophages were depleted by clodronate liposomes, as these cells have been proven to produce a large amount of IL1β.^(^ ^44^ ^)^ As revealed in Fig. 2*C*, the expression level of IL1β was remarkably decreased in the mice depleted of macrophages on days 3 and 7 post‐injury, suggesting that macrophages primarily contribute to IL1β production.

Next, we investigated whether the polarized phenotype of macrophages in tibial defect tissue with an impaired transition from the M1‐like (pro‐inflammatory) to M2‐like (anti‐inflammatory) phenotype could contribute to chronic inflammation and delayed bone repair. As found in Fig. 2*D–G* and Supplemental Fig. S2*A*, *B*, there was no significant difference in the infiltration of F4/80^+^ macrophages in indicated groups at three time points (3, 7, and 10 days) post‐injury. A relatively upregulated M1‐like macrophage (F4/80^+^ and iNOS^+^) proportion on days 3 and 7 were observed in the GIT1 CKO mice. However, the proportion of M2‐like macrophages (F4/80^+^ and CD206^+^) was not significantly altered at three time points post‐injury. Collectively, these results indicated that macrophage GIT1 plays an important role in controlling IL1β production and the M1‐like macrophage phenotype polarization on days 3 and 7 during IO.

**Fig. 3.**
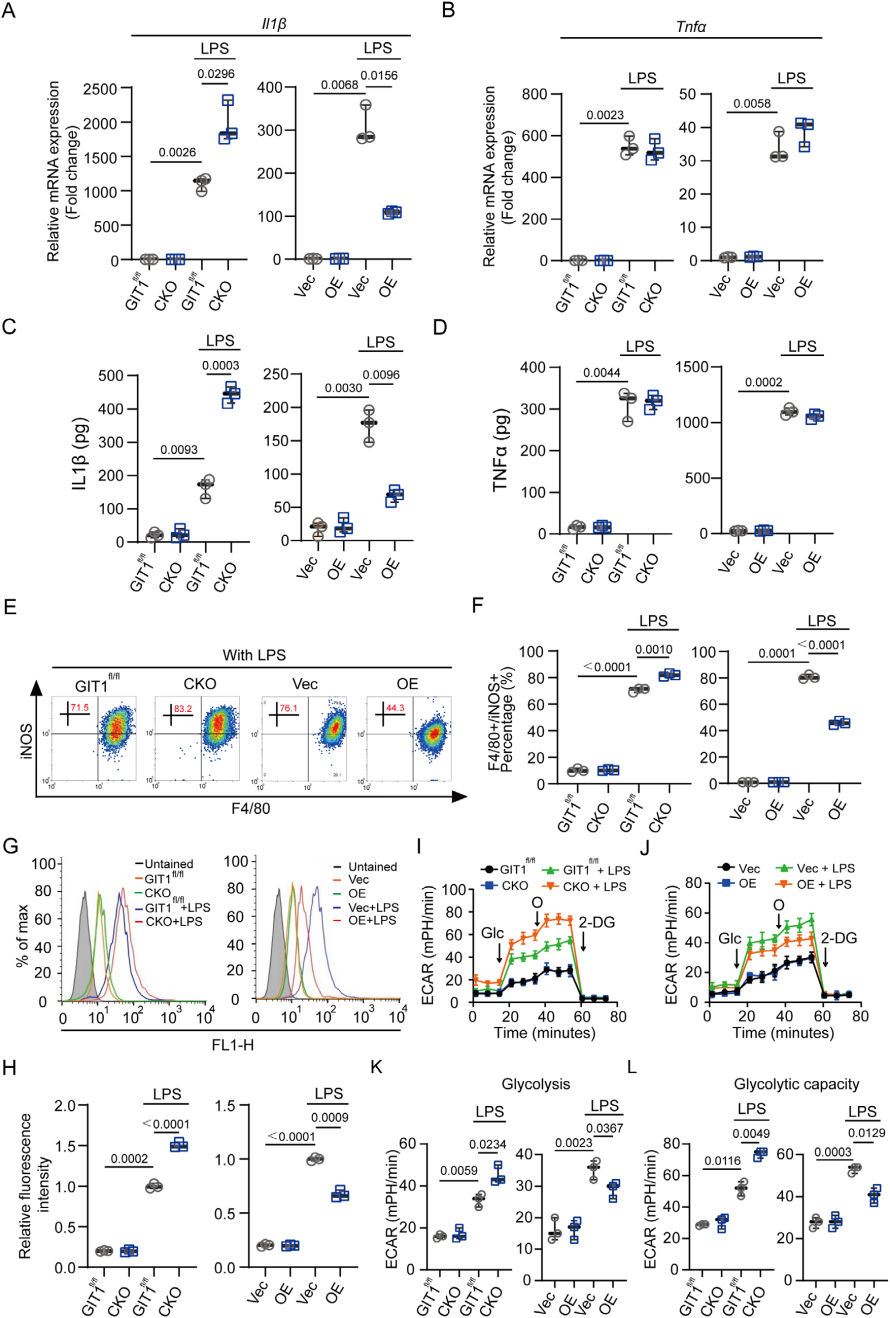
Macrophage GIT1 modulates the pro‐inflammatory response to LPS in vitro. (*A*, *B*) mRNA expression levels of IL1β (*A*) and TNFα (*B*) in BMDMs (GIT1^fl/fl^ versus CKO) and RAW264.7 (Vec versus OE) cells from different groups with or without LPS treatment were detected by qPCR (two‐way ANOVA with post hoc test). (*C*, *D*) ELISA was used to determine the secreted IL1β (*C*) and TNFα (*D*) in a serum‐free conditioned medium from macrophages in different groups (GIT1^fl/fl^ versus CKO, Vec versus OE) with or without LPS treatment (two‐way ANOVA with post hoc test). (*E*) Flow cytometry analysis of GIT1^fl/fl^ and CKO BMDMs, and Vec and OE RAW264.7 cells in response to LPS. Dot plots represent F4/80 and iNOS staining. (*F*) Percentages of M1‐like (F4/80^+^ and iNOS^+^) macrophages with or without LPS treatment were accessed (two‐way ANOVA with post hoc test). (*G*) Analysis of ROS production by flow cytometry in GIT1^fl/fl^ and CKO BMDMs (left panel) and Vec and OE RAW264.7 cells (right panel) with or without LPS treatment. (*H*) Quantification of ROS production in BMDMs and RAW264.7 cells of indicated groups (two‐way ANOVA with post hoc test). (*I*, *J*) ECAR measurement in GIT1^fl/fl^ and CKO BMDMs (*I*), and Vec and OE RAW264.7 cells (*J*) with sequential addition of glucose (Glc), oligomycin (O), and 2‐deoxyglucose (2‐DG) via a seahorse bioscience XFp analyzer. (*K*, *L*) ECAR quantification of glycolysis (*K*) and glycolytic capacity (*L*) were determined in indicated groups (two‐way ANOVA with post hoc test). ROS = reactive oxygen species; ECAR = extracellular acidification rate.

We then investigated the above effects of GIT1 on macrophages in vitro. As shown in Supplemental Fig. S3*A*, BMDMs were differentiated normally, and comparable amounts of BMDMs were obtained from both the GIT1 CKO and GIT1^fl/fl^ mice, suggesting that GIT1 knockout does not affect the differentiation or maturation of BMDMs. In accordance with our previous results, the OC formation was not affected in either group (Supplemental Fig. S3*B*). Furthermore, GIT1‐overexpressing (GIT1 OE) RAW264.7 cells were generated and confirmed by Western blotting (Supplemental Fig. S3*C*). As expected, significant upregulation of IL1β was found in LPS‐treated GIT1 CKO BMDMs, but this gene was dramatically decreased in LPS‐treated GIT1 OE RAW264.7 cells (Fig. 3*A*). {FIG3} Similar results were observed in LPS‐activated BMDMs and RAW264.7 cells from different groups via ELISA (Fig. 3*C*). In contrast, the expression levels of another pro‐inflammatory gene (TNFα) were not changed in BMDMs and RAW264.7 cells when GIT1 was depleted or overexpressed with or without LPS activated (Fig. 3*B*, *D*). As shown in Fig. 3*E*, *F* and Supplemental Fig. S3*D*, compared with the relative control group, higher percentages of F4/80^+^ iNOS^+^ BMDMs were detected in the LPS‐treated GIT1 CKO group, whereas a lower portion of F4/80^+^ iNOS^+^ cells was observed in the LPS‐treated GIT1 OE group. Moreover, the levels of LPS‐induced ROS, that is, another characteristic of M1‐like macrophages, were boosted by GIT1 depletion and limited via GIT1 overexpression (Fig. 3*G*, *H*). There is rapidly growing interest on the relationship between the metabolic reprogramming and cellular function of macrophages. For macrophages, a switch to glycolysis during LPS activation is needed to support anabolic pathways and biosynthesis.^(^ ^45^ ^)^ To determine the functional role of GIT1 in this process, we examined the modulations of glycolytic metabolic levels in GIT1^fl/fl^ and GIT1 CKO BMDMs, and Vec and OE RAW264.7 cells in both the presence and absence of LPS stimulation (Fig. 3*I–L*). The results clearly showed that compared with GIT1^fl/fl^ BMDMs, there was a significantly higher glycolysis and glycolytic capacity in the GIT1 CKO BMDMs, whereas quantification of the glycolysis and glycolytic capacity revealed a decrease in the GIT1 OE RAW264.7 cells (Fig. 3*I–L*). However, the above effects were diminished in the non‐activated BMDMs and RAW264.7 cells from all groups (Fig. 3*I–L*). These data suggest that macrophage GIT1 could regulate the pro‐inflammatory response to LPS in vitro.

### GIT1‐deficient BMDMs negatively regulate the osteoinductive effect for BMSCs by IL1β

**Fig. 4.**
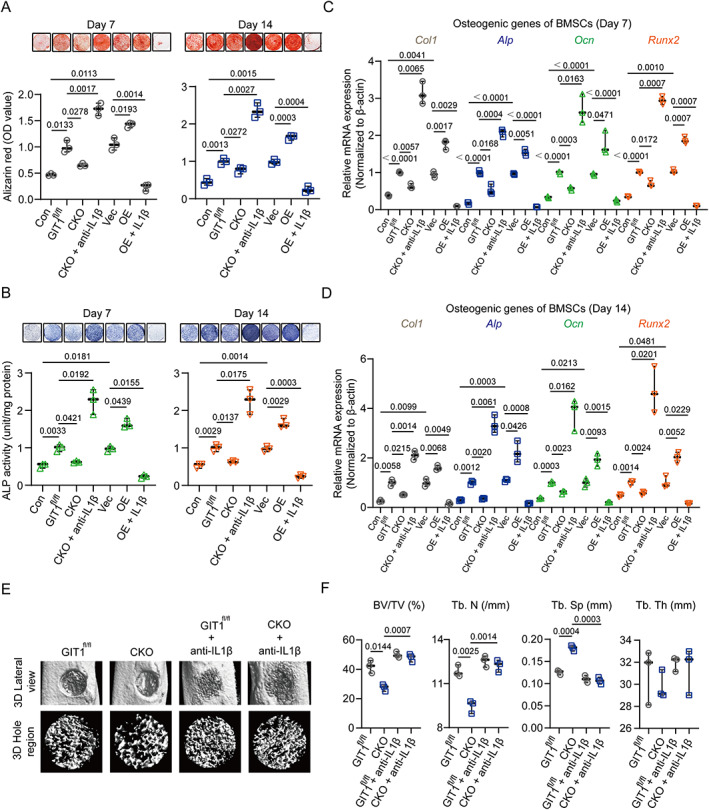
Macrophage GIT1 regulates BMSC osteogenic differentiation by modulating IL1β production. (*A*, *B*) BMSCs were cultured in an osteogenesis induction medium plus CM from LPS‐activated macrophages (BMDMs and RAW264.7 cells) from indicated treatment. BMSCs cultured in osteoblast differentiation medium alone were defined as the control group (con). After 7 and 14 days, matrix mineralization was determined via AR staining (top panel of *A*) and ALP staining (top panel of *B*). Quantitative analyses of AR staining (lower panel of *A*) and ALP activities (lower panel of *B*) on days 7 and 14 are shown (two‐way ANOVA with post hoc test). (*C*, *D*) After 7 and 14 days, mRNA expression levels of osteoblast‐specific genes (*Col1*, *Alp*, *Ocn*, and *Runx2*) of indicated groups were detected by qPCR. β‐Actin was used as an internal control (two‐way ANOVA with post hoc test). (*E*) Representative 3D reconstruction images of injured tibias (top panel) and mineralized callus (lower panel) in the defect area of GIT1^fl/fl^ and GIT1 CKO mice with or without anti‐IL1β blocking antibody treatment. (*F*) BV/TV (%), Tb.N, Tb.Sp, and Tb.Th of the mineralized bone formed in the defect region were analyzed using micro‐CT (two‐way ANOVA with post hoc test). CM = conditioned media; AR = alizarin red; ALP = alkaline phosphatase.

Next, we determined whether the negative impact of GIT1‐depleted BMDMs on the osteogenic differentiation capacities of BMSCs is due to the increased IL1β production. To start, in agreement with previously published evidence,^(^ ^32^ ^)^ we observed that IL1β inhibited the osteoblastic differentiation of BMSCs via adding recombined mouse IL1β directly (Supplemental Fig. S3*E*). When BMSCs were seeded in the presence of osteoblast differentiation medium and GIT1^fl/fl^ CM, their osteogenic differentiation ability was enhanced compared with those treated with osteoblast differentiation medium alone on days 7 and 14 via alizarin red staining and an ALP enzyme assay (Fig. 4*A*, *B*). {FIG4} However, this increased osteoinductive effect was largely limited in the BMSCs when cultured in osteoblast differentiation medium and CKO CM (Fig. 4*A*, *B* and Supplemental Fig. S3*F*). Furthermore, this limited effect of CKO CM on the BMSCs could be drastically blocked via using an anti‐IL1β monoclonal antibody (Fig. 4*A*, *B* and Supplemental Fig. S3*F*). Compared with the vector group, a more pro‐osteogenic differentiation effect was observed in the BMSCs after they were cultured in osteoblast differentiation medium and CM from LPS‐activated GIT1‐overexpressing RAW264.7 cells (OE CM) (Fig. 4*A*, *B* and Supplemental Fig. S3*F*). However, additional stimulation with IL1β severely inhibited the osteogenic differentiation of the BMSCs in the OE CM group (Fig. 4*A*, *B* and Supplemental Fig. S3*F*). The quantification of the mRNA expression levels of osteogenic marker genes (collagen type I [*Col1*], *Alp*, osteocalcin [*Ocn*], and runt‐related transcription factor 2 [*Runx2*]) on days 7 and 14 in these groups also supported the above results (Fig. 4*C*, *D*). Notably, compared with the GIT1^fl/fl^ CM, CKO CM did not alter the proliferation ability of BMSCs in vitro (Supplemental Fig. S3*G*). It might suggest that macrophage GIT1 did not affect the proliferation of BMSCs in vitro. Furthermore, we tested whether blocking IL1β would impact bone regeneration in vivo. The 3D reconstruction images and morphometric parameter analysis data (drastically increased BV/TV and Tb.N, and decreased Tb.Sp) indicated that anti‐IL1β blocking antibody treatment could significantly enhance the bone regeneration in GIT1 CKO mice (Fig. 4*E*, *F*). Collectively, these results indicated that macrophage GIT1 takes part in modulating the osteoinductive effect for BMSCs via IL1β secretion regulation, which might reinforce the theory that GIT1‐depleted macrophages contributed to the delayed bone regeneration via enhancing IL1β production.

### Role of GIT1 in regulating NRF2 activity in LPS‐activated macrophages

**Fig. 5.**
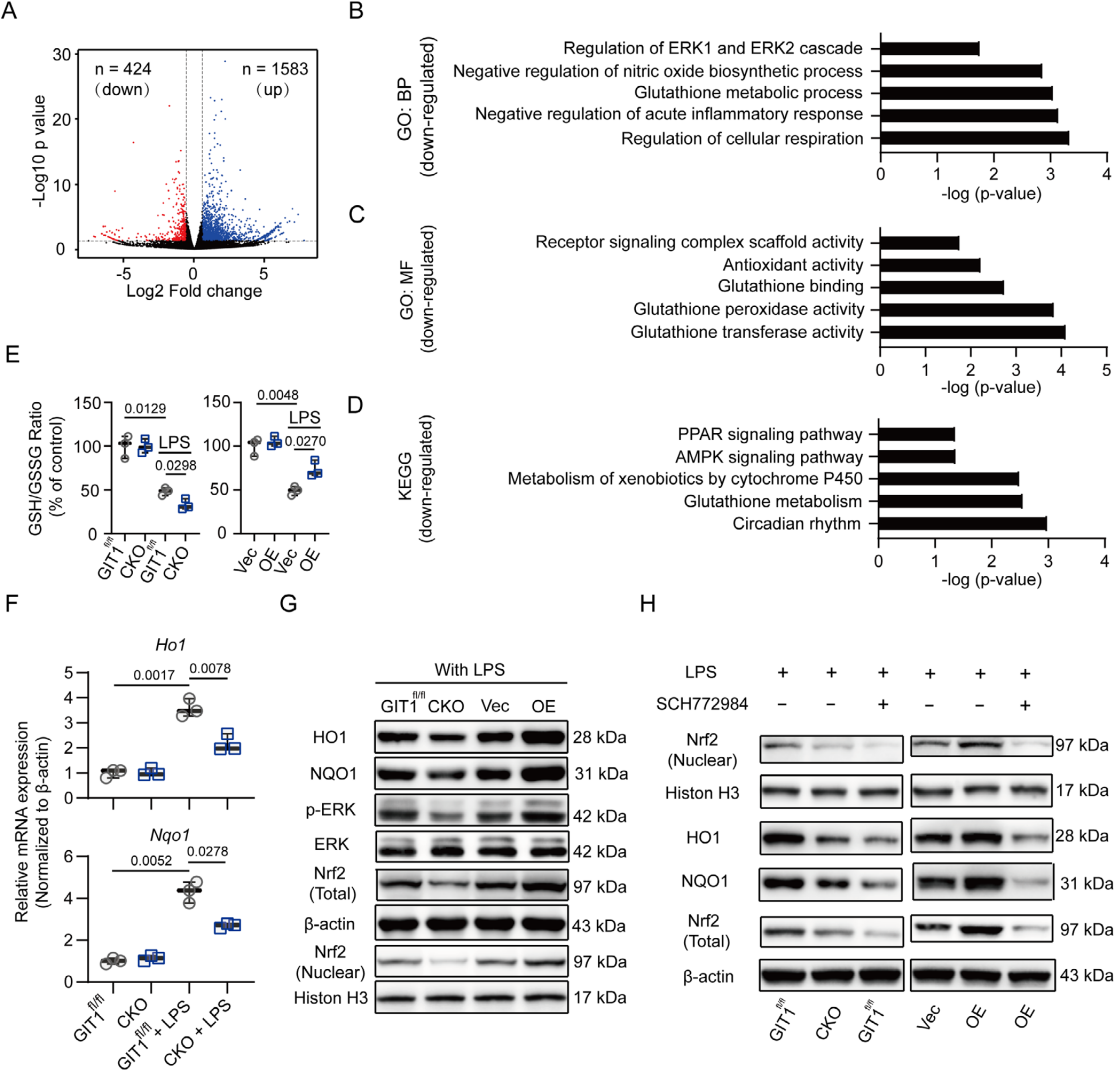
Macrophage GIT1 activates NRF2 by phosphorylating ERK in response to LPS. (*A*) Volcano plot of genes of GIT1^fl/fl^ and CKO BMDMs in response to LPS. Blue and red dots represent up‐ and downregulated DEGs, respectively. (*B–D*) Representative BP (*B*) and MF (*C*) categories using GO analyses and KEGG pathways (*D*) based on downregulated DEGs affected by GIT1 depletion in BMDMs after LPS treatment. (*E*) Determination of the GSH/GSSG ratio in BMDMs and RAW264.7 cells of indicated groups (two‐way ANOVA with post hoc test). (*F*) mRNA expressions of NRF2 target genes (*Ho1* and *Nqo1*) in BMDMs of GIT1^fl/fl^ and CKO groups with or without LPS were detected using qPCR. GAPDH was used as an internal control (two‐way ANOVA with post hoc test). (*G*) Western blotting indicated the altered protein expression levels of HO1, NQO1, p‐ERK/ERK, NRF2 (nuclear), and NRF2 (total) in indicated groups of macrophages (BMDMs and RAW264.7 cells) after LPS treatment. (*H*) Western blotting analysis of NRF2 (nuclear) and NRF2 (total) in BMDMs of indicated groups treated with SCH772984 (an ERK inhibitor). BP = biological process; MF = molecular function; GO = gene ontology; KEGG = Kyoto encyclopedia of genes and genomes; GSH = reduced glutathione; GSSG = oxidized glutathione.

To explore the mechanism of the aforementioned functions in GIT1‐depleted macrophages on the LPS response, we performed a transcriptome analysis via high‐throughput RNA sequencing (RNA‐seq), including three biological replicates of GIT1 CKO and GIT1^fl/fl^ BMDMs after LPS treatment. As shown in Fig. 5 *A*, {FIG5} via a volcano plot, compared with the GIT1^fl/fl^ group, a total of 1583 DEGs were upregulated and 424 DEGs were downregulated in the GIT1 CKO group. As expected, the GO and KEGG pathway analysis based on 429 downregulated DEGs indicated that several antioxidant activities and anti‐inflammatory responses were involved (Fig. 5*B–D*). In accordance with the widely accepted view that NRF2 is a central player in redox control and inflammation limitation in LPS‐activated macrophages,^(^ ^18^, ^19^, ^20^ ^)^ we next investigated the mechanisms by which GIT1 might regulate NRF2 in LPS‐treated macrophages. The knockout of GIT1 in the BMDMs decreased the GSH/GSSG ratio and downregulated the expression level of two NRF2 target genes, HO1 and NQO1, in response to LPS (Fig. 5*E–G* and Supplemental Fig. S4*A*, *B*). Additionally, an increased GSH/GSSG ratio and enhanced expression level of HO1 and NQO1 were observed in GIT1‐overexpressing RAW264.7 cells after LPS treatment (Fig. 5*E*, *G* and Supplemental Fig. S4*A*, *B*). Several recent studies have highlighted the involvement of ERK in the activation of NRF2.^(^ ^16^, ^46^ ^)^ Because GIT1 is essential for the activation of ERK,^(^ ^24^, ^25^, ^26^ ^)^ we tested whether macrophage GIT1 activates NRF2 through ERK in response to LPS. As shown in Fig. 5*G* and Supplemental Fig. S4*A*, *B*, the protein levels of phosphorylated ERK, total NRF2, and nuclear‐localized NRF2 were all affected by GIT1 in LPS‐stimulated macrophages (BMDMs and RAW264.7 cells) yet had no effect without LPS treatment. A small molecule inhibitor targeting ERK (SCH772984) was further used to verify the role of ERK in GIT1‐mediated NRF2 activation. In Fig. 5*H* and Supplemental Fig. S4*C*, in response to LPS, weaker bands of HO1, NQO1, and total and nuclear‐localized NRF2 were observed in the GIT1^fl/fl^ and GIT1‐overexpressing groups when treated with SCH772984. These results suggested that macrophage GIT1 could activate NRF2 through ERK1/2 in response to LPS.

### Macrophage GIT1 controlled IL1β production and glycolysis in an ERK/NRF2‐dependent manner

**Fig. 6.**
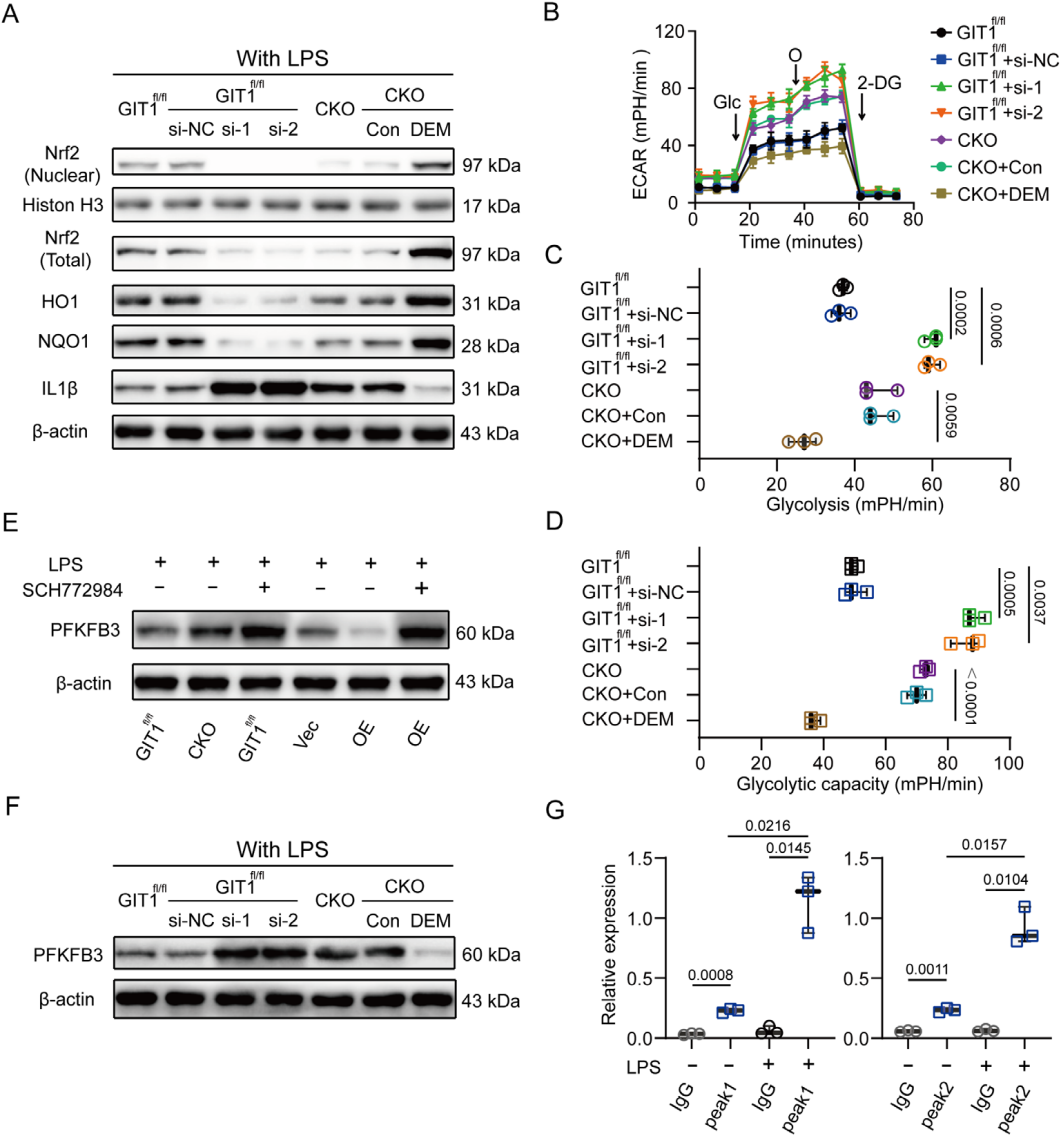
IL1β production and glycolysis of macrophage in response to LPS were governed by GIT1 in an ERK/NRF2‐dependent manner. (*A*) Immunoblot images showing protein levels of NRF2 (nuclear), NRF2 (total), HO1, NQO1, IL1β, and β‐Actin of BMDMs in indicated groups. NRF2‐siRNA (si‐1 and si‐2) could effectively increase the expression level of IL1β proteins, and DEM‐treated cells limited IL1β production. (*B*) LPS‐induced ECAR in BMDMs after treatment with NRF2‐siRNA or DEM, as indicated. (*C*, *D*) Quantification of glycolysis (*C*) and glycolytic capacity (*D*) were revealed in indicated groups (one‐way ANOVA with post hoc test). (*E*) In response to LPS, altered protein expression levels of PFKFB3 in BMDMs and RAW264.7 cells when blocking ERK using SCH772984 were detected via Western blotting. (*F*) After treated with LPS, the protein expression patterns of PFKFB3 in GIT1^fl/fl^ BMDMs with or without NRF2 silencing and GIT1 CKO BMDMs with or without DEM treatment were shown. (*G*) CHIP‐qPCR analysis of NRF2‐binding regions (peaks 1 and 2) in GIT1^fl/fl^ BMDMs with or without LPS treatment (two‐way ANOVA with post hoc test). DEM = diethyl maleate; CHIP‐qPCR = chromatin immunoprecipitation with quantitative polymerase chain reaction.

Recently, NRF2 was linked to the direct regulation of IL1β production in macrophages.^(^ ^18^, ^19^, ^20^ ^)^ Therefore, we investigated whether macrophage GIT1 mediated IL1β production in an ERK/NRF2‐dependent manner. First, SCH772984 was used to confirm that macrophage GIT1‐mediated ERK phosphorylation is able to regulate IL1β production in response to LPS (Supplemental Fig. S5*A*). Since NRF2 is able to regulate ROS production, we then figured out whether the GIT1‐mediated IL1β limitation depends on the ROS levels. As shown in Supplemental Fig. S5*B*, NAC as used to treat BMDMs and RAW264.7 cells in the indicated groups. LPS treatment enhanced the production of IL1β in GIT1 CKO BMDMs and reduced IL1β level in GIT1 OE RAW264.7 cells. NAC treatment did not affect the IL1β production in the indicated BMDMs and RAW264.7 cells after treated with LPS (Supplemental Fig. S5*B*), suggesting that GIT1‐mediated controlling is independent of ROS control. Then, in Fig. 6*A* {FIG6} and Supplemental Fig. S5*C*, when NRF2 was silenced using siRNA in GIT1^fl/fl^ BMDMs, the protein level of IL1β was dramatically upregulated in response to LPS. Moreover, pharmacological activation of NRF2 in LPS‐treated GIT1 CKO BMDMs using small DEM effectively limited the IL1β production (Fig. 6*A* and Supplemental Fig. S5*C*).

It is known that M1‐like macrophages are characterized by increased glycolysis, which enables the cells to cope with the high energy demand.^(^ ^13^ ^)^ Thus, we further explored whether GIT1 indeed acts through ERK/NRF2 to modulate glycolysis. As revealed in Fig. 6*B–D*, the ECAR was significantly enhanced after silencing NRF2 in LPS‐activated GIT1^fl/fl^ BMDMs. Moreover, we tested the consequences of the chemical induction of NRF2 in GIT1 CKO BMDMs in response to LPS. The results showed that the accumulated NRF2 reversed the effect of the GIT1 depletion, reducing the glycolysis and glycolytic capacity (Fig. 6*B–D*). Previous reports have indicated that PFKFB3, a critical factor that controls glycolysis, is regulated by NRF2 in human umbilical vein endothelial cells.^(^ ^47^ ^)^ To determine the regulatory role of GIT1 in PFKFB3 in macrophages, we compared the expression patterns of PFKFB3 in GIT1^fl/fl^ and GIT1 CKO BMDMs in response to LPS. The results of the RNA‐seq, qPCR, and Western blotting analyses all revealed that the GIT1 deficiency increased the expression of PFKFB3 after treated with LPS (Supplemental Fig. S5*D–F*). However, the GIT1 overexpression was observed to decrease the level of PFKFB3 in the indicated RAW264.7 cells in response to LPS (Supplemental Fig. S5*E–G*). We then investigated whether macrophage GIT1‐mediated ERK/NRF2 activation regulates PFKFB3. As shown in Fig. 6*E* and Supplemental Fig. S5*H*, blocking ERK in GIT1^fl/fl^ BMDMs and GIT1 OE RAW264.7 cells reversed the reduced expression of PFKFB3. Furthermore, in Fig. 6*F* and Supplemental Fig. S5*I*, NRF2 silencing via siRNA reversed the expression of PFKFB3 in GIT1^fl/fl^ BMDMs, and induced NRF2 expression was shown to inhibit PFKFB3 in GIT1 CKO BMDMs. To gain further insights into the relationship of NRF2 and PFKFB3, bioinformatics analyses via IVG based on online CHIP‐sequence data (GSE 36030) suggested two potential NRF2 binding sites on the regulatory regions of PFKFB3 (Supplemental Fig. S5*J*). Subsequently, a CHIP‐qPCR was performed to confirm the binding of NRF2 to the selected regions of PFKFB3. As shown in Fig. 6*G*, NRF2 binding to PFKFB3 loci was observed in both untreated and LPS‐activated cells, and a higher NRF2‐binding signal was observed in LPS‐stimulated BMDMs. Together, these results show that the GIT1/ERK/NRF2 axis regulated macrophage IL1β production and PFKFB3 expression. The regulatory effect of GIT1 in macrophage glycolysis might explain the finding that GIT1 knockout increased the fraction of M1‐like macrophages.

### Transplantation of GIT1^fl^ ^/fl^ bone marrow improved the regeneration of bone defects in the GIT1 CKO mice

**Fig. 7.**
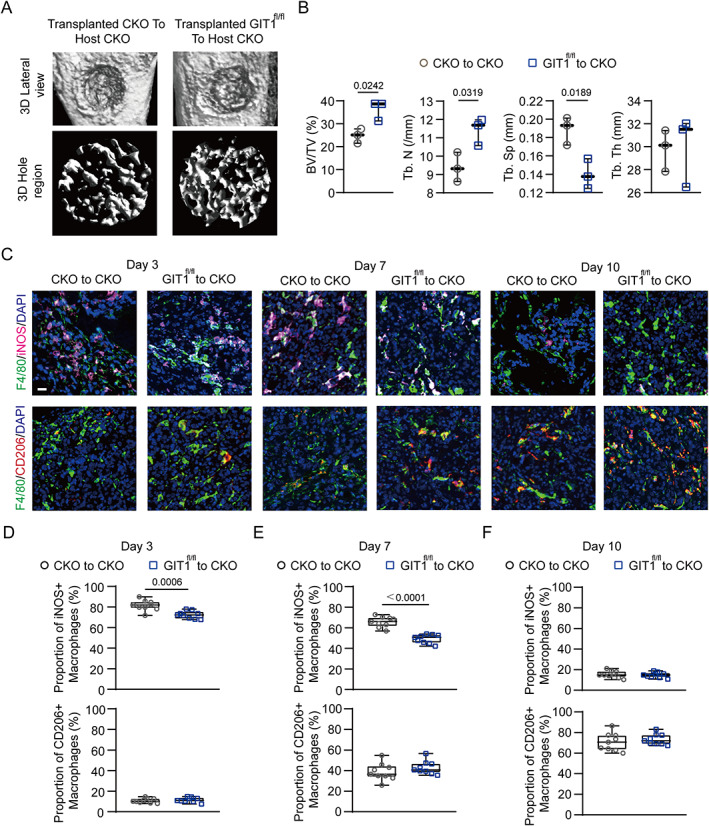
Substitution of GIT1 CKO bone marrow with GIT1^fl/fl^ bone marrow facilitates intramembranous bone healing. (*A*) Micro‐CT reconstruction of tibial defect (top panel) and mineralized bone in the hole region (lower panel) of indicated groups. (*B*) Quantitative analysis of BV/TV (%), Tb.N, Tb.Sp, and Tb.Th of the regenerated bone in the tibial defect from different groups (unpaired two‐tailed Student's *t* test). (*C*) Infiltration of M1‐like (F4/80^+^ and iNOS^+^) (top panel) and M2‐like (F4/80^+^ and CD206^+^) (lower panel) macrophages in the tibial defect region from indicated groups on days 3, 7, and 10 post‐injury were identified using IF staining. Nuclei were counterstained with DAPI (blue). Scale bar = 100 μm. (*D–F*) Proportion of infiltrated M1‐like (F4/80^+^ and iNOS^+^) and M2‐like (F4/80^+^ and CD206^+^) macrophages at indicated time points post‐injury from different transplanted mice (CKO to CKO versus GIT1^fl/fl^ to CKO) were determined (unpaired two‐tailed Student's *t* test).

To further confirm the linkage between GIT1 in myeloid macrophages and IO, bone marrow transplantation (BMT) assays were conducted in which irradiated GIT1 CKO mice were reconstituted with bone marrow from either GIT1 CKO or GIT1^fl/fl^ mice. As shown in Fig. 7*A* {FIG7 }and Supplemental Fig. S5*A*, compared with the relative control mice, more newly formed bone tissue on day 7 post‐injury was observed in the GIT1 CKO mice transplanted with GIT1^fl/fl^ bone marrow via micro‐CT imaging and histological analyses. As expected, increased BV/TV and Tb.N values and a decreased Tb.Sp value were found in the irradiated GIT1 CKO mice reconstituted with GIT1^fl/fl^ bone marrow (Fig. 7*B*). In addition, these irradiated GIT1 CKO mice contributed to a decreased proportion of iNOS^+^ macrophages on days 3 and 7 post‐injury (Fig. 7*C–F* and Supplemental Fig. S5*B*). Decreased production of IL1β on days 3 and 7 post‐injury was also observed in the GIT1 CKO mice after the GIT1^fl/fl^ bone marrow transplantation (Supplemental Fig. S5*C*). These results reinforced the findings that macrophage GIT1 is a key regulator of IO by affecting the production of IL1β and proportion of infiltrated M1‐like macrophages.

## Discussion

**Fig. 8.**
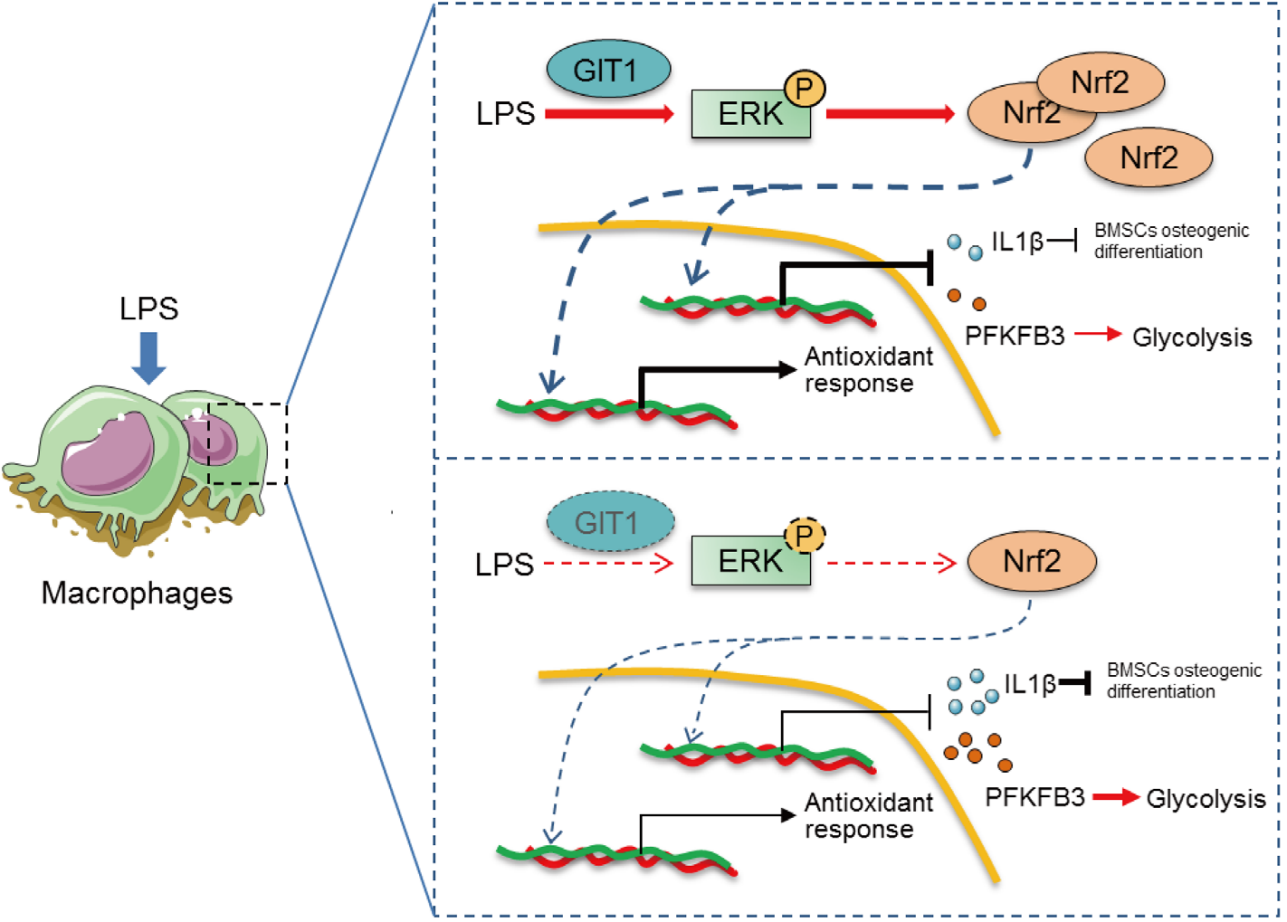
Macrophage GIT1 is critical to successful bone regeneration via regulating inflammatory responses in an ERK/NRF2‐dependent way. In response to LPS, once GIT1 is involved, it promotes NRF2 to stabilize and translocate into the nucleus for genes transcriptionally regulation via phosphorylating ERK. In the absence of GIT1, inactivated ERK/NRF2 axis could lead to abnormal upregulation of ROS production and IL1β secretion and glycolysis, which is detrimental to the osteogenic differentiation of BMSCs. Furthermore, macrophage GIT1‐mediated activation of ERK/NRF2 controls glycolysis by limiting PFKFB3 expression.

A growing amount of evidence has established that optimal fracture repair relies on a well‐orchestrated interplay between inflammatory response and MSCs.^(^ ^7^, ^8^, ^41^ ^)^ While the roles of macrophages in the regulation of the inflammatory response and bone regeneration during fracture healing are increasingly being recognized, the molecular bases of these functions remain to be elucidated. The most significant finding of the present study is that macrophage GIT1 is critical to successful bone regeneration both in vivo and in vitro. Our data further uncovered that the macrophage GIT1/ERK/NRF2 axis is the key regulator of redox homeostasis, IL1β production, and glycolysis, providing an explanation as to why macrophages lacking GIT1 are highly pro‐inflammatory and detrimental to the osteogenic differentiation of BMSCs in response to LPS (Fig. 8). {FIG8} Collectively, our data provide a novel mechanistic basis for the role of macrophage GIT1 in IO.

Importantly, the regenerative capacities of endogenous and/or transplanted stem cells are based on the specific microenvironment at the bone defect area. Recently, a number of studies have indicated that a series of cytokines released from M1‐like macrophages are responsible for the regulation of MSC osteogenesis.^(^ ^41^, ^48^, ^49^, ^50^ ^)^ Oncostatin M and prostaglandin E2 (PGE2) have been proven beneficial for the increased osteogenesis of MSCs.^(^ ^49^, ^50^ ^)^ However, paradoxical results have been reported regarding the effects of IL1β on the osteogenic differentiation of MSCs.^(^ ^51^ ^)^ Someone concluded that IL1β facilitates this osteogenic differentiation in an inflammatory microenvironment.^(^ ^52^ ^)^ In contrast, the majority of studies have proposed that IL1β inhibits the osteogenesis of MSCs through canonical NF‐κB signaling as well as other pathways such as IL1R1/MyD88 signaling.^(^ ^32^, ^53^, ^54^ ^)^ This controversy might be partially explained by technical differences among the research works, such as different cell sources, stimulation conditions, and IL1β doses. In our study, the aberrant secretion of IL1β in GIT1‐depleted macrophages primarily caused impaired bone regeneration. Moreover, except for regulating IL1β transcription, increasingly studies investigated the processing of the inactive IL1β precursor into the bioactive IL1β.^(^ ^55^ ^)^ The activation of caspase‐1 by inflammasomes was proven to be involved in the processing of bioactive pro‐IL1β.^(^ ^55^ ^)^ Therefore, whether GIT1 contributes to the processing of pro‐IL1β is worthy to be explored further.

Previous studies have determined that NRF2 is a master transcriptional regulator of the expression of genes that govern various cytoprotective functions, such as (i) antioxidant activity, (ii) drug metabolism, (iii) anti‐inflammatory responses, and (iv) cellular metabolism.^(^ ^15^, ^16^ ^)^ Despite the high amount of support for the regulation of NRF2 via Keap1, a body of evidence holds that NRF2 can be regulated independently.^(^ ^16^, ^21^ ^)^ In our study, we determined that GIT1‐mediated ERK phosphorylation is responsible for the activation of NRF2. However, the exact phosphorylated site has yet to be identified. Although NRF2 has traditionally been regarded as an activating transcription factor, the transcription‐inhibiting effect of NRF2 in M1‐like macrophages is being increasingly recognized.^(^ ^18^, ^19^, ^20^ ^)^ Several recent studies support the viewpoint that NRF2 displays an anti‐inflammatory role in an ROS‐independent manner by directly inhibiting the transcriptional activities of several inflammation‐related genes, such as IL1α, IL1β, and IL6, in M1‐like BMDMs.^(^ ^18^, ^19^, ^20^ ^)^ Our present findings indicate the presence of a limited expression pattern of IL1β attributed to GIT1‐mediated NRF2 activation.

It is generally accepted that cellular metabolism reprograms are the key to determining the polarized phenotypes and functions of macrophages.^(^ ^45^ ^)^ Moreover, the existence of a connection between GIT1 and cellular metabolism has been established, as GIT1 has been reported to be a regulator of mitochondrial biogenesis and function in the heart.^(^ ^56^ ^)^ However, the relevance of GIT1 in the process by which LPS treatment triggers a shift toward a glycolytic metabolic state in macrophages has not been explored. Here, for the first time, we report on the functional role of the macrophage GIT1/ERK/NRF2 axis in the process of glycolysis in response to LPS. Furthermore, PFKFB3, a critical control point during glycolysis, is confirmed as a critical target gene for the GIT1/ERK/NRF2 axis in this study. However, in contrast to previous data on transcriptional activation by NRF2, the transcriptional capacity was inhibited by NRF2 in LPS‐simulated BMDMs here. The precise molecular mechanism of how NRF2 induces the inhibition of transcriptional regulation to PFKFB3 in M1‐like BMDMs is complicated and, thus, the focus of our ongoing investigation.

In summary, we provide insights into the regulatory effect and specific mechanism of GIT1 during bone regeneration. Macrophage GIT1 is essential for bone repair during IO. Furthermore, the results of our function experiments suggest that macrophage GIT1‐mediated NRF2 activation controls antioxidant activity, IL1β production, and glycolysis in response to LPS. These findings are a turning point in the understanding of GIT1 functions related to bone regeneration. Our work may provide a basis for therapeutic strategies based on GIT1 modulation to improve bone repair and regeneration.

## Disclosures

All authors state that they have no conflicts of interest.

## Peer Review

The peer review history for this article is available at https://publons.com/publon/10.1002/jbmr.4099.

## Supporting information


**Supplemental Fig. S1.** (*A*) GIT1^fl/fl^; Lyz2‐Cre^+^ offspring were crossed with each other, genomic DNA was extracted from tails, and genotyping was detected by PCR. GIT1^fl/fl^ Lyz2‐Cre mice were considered myeloid‐specific GIT1 CKO mice and were named CKO. P = positive control; B6 = genomic DNA from B6 mice (negative control); N = no‐template control. (*B*) Expression patterns of GIT1 in several kinds of primary cells from GIT1^fl/fl^ and GIT1 CKO groups were identified using Western blotting. Densitometric analysis showed the relative amounts of GIT1 in the indicated groups (lower panel). OB = osteoblast; BMSC = bone marrow stromal cell; Chod = chondrocyte; OC = osteoclast; BMDM = bone marrow‐derived macrophage. (Left panel: one‐way ANOVA with post hoc test; right panel: two‐way ANOVA with post hoc test). (*C*) Schematic representing the experimental design of OPG, CLOD, and the relative control regent injection regimen, and tissue harvest. OPG = osteoprotegerin; CLOD = clodronate liposomes; Con lip = control liposome. (*D*) Number of TRAP^+^ cells in the injury region using TRAP staining revealed a significant decrease in TRAP^+^ cells after OPG treatment in both GIT1^fl/fl^ and GIT1 CKO groups. Scale bar = 100 μm. (*E*) After treatment with clodronate liposomes or control liposomes, representative flow cytometry images indicated the percentage of CD11b^+^ cells in peripheral blood in GIT1^fl/fl^ and GIT1 CKO mice. (*F*) Quantification of the percentage of CD11b^+^ cells in the indicated groups suggested that the majority of monocytes/macrophages are depleted with CLOD (one‐way ANOVA with post hoc test). (*G*) Representative images of micro‐CT reconstruction of injured tibias, mineralized callus, and H&E staining in the defect area of indicated groups. Scale bar = 100 μm.
**Supplemental Fig. S2.** (*A*, *B*) Infiltrated F4/80^+^ macrophages were determined at indicated time points postoperatively from GIT1^fl/fl^ and GIT1 CKO mice using IF staining. iNOS group (*A*) revealed the tissues stained with anti‐F4/80 and anti‐iNOS; samples stained with anti‐F4/80 and anti‐CD206 defined as the CD206 group (*B*). Statistical analysis: unpaired two‐tailed Student's *t* test.
**Supplemental Fig. S3.** (*A*) Representative images of BMDMs derived from GIT1^fl/fl^ and GIT1 CKO mice (left panel). Scale bar = 100 μm. Flow cytometry analysis was used to determine F4/80 expression patterns in different groups (right panel). (*B*) Representative pictures of TRAP^+^ osteoclasts obtained from GIT1^fl/fl^ and GIT1 CKO mice (left panel). Scale bar = 100 μm. The number of osteoclasts in GIT1^fl/fl^ and GIT1 CKO groups were identified using TRAP staining (right panel) (unpaired two‐tailed Student's *t* test). (*C*) Overexpression efficacy of GIT1 in RAW264.7 cells in indicated groups was confirmed using Western blotting. Densitometric analysis showed the relative amounts of GIT1 in the indicated groups (upper panel) (two‐way ANOVA with post hoc test). (*D*) Flow cytometry analysis of GIT1^fl/fl^ and CKO BMDMs, and Vec, and OE RAW264.7 cells in the absence of LPS. Dot plots represent F4/80 and iNOS staining. (*E*) BMSCs were cultured in an osteogenesis induction medium with (0.1, 0.5, and 1 ng/mL) or without IL1β. After 14 days, matrix mineralization was detected using alizarin red staining (upper paned) and quantitative analyses of AR staining was also performed (lower paned) (one‐way ANOVA with post hoc test). (*F*) Pixel quantitation of ALP images on days 7 and 14 are shown (two‐way ANOVA with post hoc test). (*G*) Knockdown of GIT1 in macrophages did not affect BMSC proliferation using the CCK‐8 assay (one‐way ANOVA with post hoc test).
**Supplemental Fig. S4.** (*A*) Protein expression levels of HO1, NQO1, p‐ERK/ERK, NRF2 (nuclear), and NRF2 (total) in indicated groups without LPS treatment were detected via Western blotting (left panel). Densitometric analysis showed the relative amounts of HO1, NQO1, p‐ERK/ERK, NRF2 (nuclear), and NRF2 (total) (right panel). (*B*) Densitometric analysis showed the relative amounts of HO1, NQO1, p‐ERK/ERK, NRF2 (nuclear), and NRF2 (total) in indicated groups after LPS treatment. (*C*) The relative amounts of HO1, NQO1, NRF2 (nuclear), and NRF2 (total) in indicated groups were measured. Statistical analysis: one‐way ANOVA with post hoc test (*A*, *B*); two‐way ANOVA with post hoc test (*C*).
**Supplemental Fig. S5.** (*A*, *B*) Western blotting was used to uncover IL1β expression patterns in the indicated groups pretreated with or without SCH772984 (*A*, lower panel) or NAC (*B*, upper panel). Also, densitometric analysis was performed to show the relative amounts of IL1β in the indicated groups. (*C*) The relative amounts of HO1, NQO1, IL1β, NRF2 (nuclear), and NRF2 (total) in indicated groups were measured. (*D*) Log2 (fold change) value of *Pfkfb1‐4* genes from RNA‐seq. (*E*, *F*) mRNA and protein levels of PFKFB3 in indicated BMDMs and RAW264.7 cells in response to LPS (*E*). Further, the relative amounts of PFKFB3 in different groups were measured (*F*). (*G*) Immunoblot image (upper panel) and densitometric analysis (lower panel) revealed the effect of macrophage (BMDM and RAW264.7 cell) GIT1 on PFKFB3 expression without LPS treatment. (*H*) Densitometric analysis showed the relative amounts of PFKFB3 in the indicated groups were performed. (*I*) The relative amounts of PFKFB3 in the indicated groups were measured. (*J*) IVG analysis of *Pfkfb3* locus showing NRF2‐binding sites (Peak1 and Peak2). Statistical analysis: one‐way ANOVA with post hoc test (*E–G*); two‐way ANOVA with post hoc test (*A–C*, *H*, *I*).
**Supplemental Fig. S6.** (*A*) Representative H&E staining images in the defect region of different transplanted mice (CKO to CKO versus GIT1^fl/fl^ to KO). Scale bar = 100 μm. (*B*) Infiltrated F4/80^+^ macrophages were quantified at different time points (days 3, 7, and 10) postoperatively from indicated mice. iNOS group (left panel): samples stained with anti‐F4/80 and anti‐iNOS; CD206 group (right panel): samples stained with anti‐F4/80 and anti‐CD206 (unpaired two‐tailed Student's *t* test). (*C*) ELISA was performed to detect the concentration of IL1β in the bone defect region at different time points (days 0, 3, 7, 10, and 14) post‐injury from different transplanted mice (CKO to CKO versus GIT1^fl/fl^ to KO) (one‐way ANOVA with post hoc test).
**Table S1.** The Sequences of Primers Used to Confirm the Genotyping of GIT1 CKO Mice
**Table S2.** The Sequences of qPCR Primers and si‐RNA Targeting NRF2
