# Supplementary material for: Macrophage GIT1 Contributes to Bone Regeneration by Regulating Inflammatory Responses in an ERK/NRF2‐Dependent Way
Source: J Bone Miner Res. 2020 Jul 9;35(10):2015–31. doi: 10.1002/jbmr.4099 (PMC7689802; doi:10.1002/jbmr.4099)

**Supplementary Figure legends**

**Supplementary Figure 1.** (a) GIT1^fl/fl^; Lyz2-Cre^+^ offspring were crossed with each other, genomic DNA was extracted from tails, and genotyping was detected by PCR. GIT1^fl/fl^ Lyz2-Cre mice were considered myeloid-specific GIT1 CKO mice and were named CKO. Abbreviation: P, positive control; B6, genomic DNA from B6 mice (negative control); N, no-template control. (b) Expression patterns of GIT1 in several kinds of primary cells from GIT1^fl/fl^ and GIT1 CKO groups were identified using western blotting. Densitometric analysis showed the relative amounts of GIT1 in the indicated groups (lower panel).Abbreviation: OB, osteoblast; BMSC, bone marrow stromal cell; Chod, chondrocyte; OC, osteoclast; BMDM, bone marrow-derived macrophage. (left panel: one-way ANOVA with post hoc test; right panel: two-way ANOVA with post hoc test) (c) Schematic representing the experimental design of OPG, CLOD and the relative control regent injection regimen, and tissue harvest. Abbreviation: OPG, osteoprotegerin; CLOD, clodronate liposomes; Con lip, control liposome. (d) Number of TRAP^+^ cells in the injury region using TRAP staining revealed a significant decrease in TRAP^+^ cells after OPG treatment in both GIT1^fl/fl^ and GIT1 CKO groups. Bar = 100 μm. (e) After treatment with clodronate liposomes or control liposomes, representative flow cytometry images indicated the percentage of CD11b^+^ cells in peripheral blood in GIT1^fl/fl^ and GIT1 CKO mice. (f) Quantification of the percentage of CD11b^+^ cells in the indicated groups suggested that the majority of monocytes/macrophages are depleted with CLOD (one-way ANOVA with post hoc test). (g) Representative images of micro-CT reconstruction of injured tibiae, mineralized callus, and H&E staining in the defect area of indicated groups. Bar = 100 μm.

**Supplementary Figure 2.** (a and b) Infiltrated F4/80^+^ macrophages were determined at indicated time points postoperatively from GIT1^fl/fl^ and GIT1 CKO mice using IF staining. iNOS group (a) revealed the tissues stained with anti-F4/80 and anti-iNOS; samples stained with anti-F4/80 and anti-CD206 defined as the CD206 group (b). Statistical analysis: unpaired two-tailed Student’s t-test.

**Supplementary Figure 3.** (a) Representative images of BMDMs derived from GIT1^fl/fl^ and GIT1 CKO mice (left panel). Scale bar = 100 µm. Flow cytometry analysis was used to determine F4/80 expression patterns in different groups (right panel). (b) Representative pictures of TRAP^+^ osteoclasts obtained from GIT1^fl/fl^ and GIT1 CKO mice (left panel). Scale bar = 100 µm. The number of osteoclasts in GIT1^fl/fl^ and GIT1 CKO groups were identified using TRAP staining (right panel) (unpaired two-tailed Student’s t-test). (c) Overexpression efficacy of GIT1 in RAW264.7 cells in indicated groups was confirmed using western blotting. Densitometric analysis showed the relative amounts of GIT1 in the indicated groups (upper panel) (two-way ANOVA with post hoc test). (d) Flow cytometry analysis of GIT1^fl/fl^ and CKO BMDMs, and Vec, and OE RAW264.7 cells in the absence of LPS. Dot plots represent F4/80 and iNOS staining. (e) BMSCs were cultured in an osteogenesis induction medium with (0.1, 0.5, and 1 ng/mL) or without IL1β. After 14 days, matrix mineralization was detected using alizarin red staining (upper paned) and quantitative analyses of AR staining was also performed (lower paned) (one-way ANOVA with post hoc test). (f) Pixel quantitation of ALP images on days 7 and 14 are shown (two-way ANOVA with post hoc test). (g) Knockdown of GIT1 in macrophages did not affect BMSCs proliferation using the CCK-8 assay (one-way ANOVA with post hoc test).

**Supplementary Figure 4.** (a) Protein expression levels of HO1, NQO1, p-ERK/ERK, NRF2 (nuclear), and NRF2 (total) in indicated groups without LPS treatment were detected via western blotting (left paned). Densitometric analysis showed the relative amounts of HO1, NQO1, p-ERK/ERK, NRF2 (nuclear), and NRF2 (total) (right paned). (b) Densitometric analysis showed the relative amounts of HO1, NQO1, p-ERK/ERK, NRF2 (nuclear), and NRF2 (total) in indicated groups after LPS treatment. (c) The relative amounts of HO1, NQO1, NRF2 (nuclear), and NRF2 (total) in indicated groups were measured. Statistical analysis: one-way ANOVA with post hoc test (a and b); two-way ANOVA with post hoc test (c).

**Supplementary Figure 5.** (a and b) Western blotting was used to uncover IL1β expression patterns in the indicated groups pretreated with or without SCH772984 (a, lower panel) or NAC (b, upper panel). Also, densitometric analysis was performed to show the relative amounts of IL1β in the indicated groups. (c) The relative amounts of HO1, NQO1, IL1β, NRF2 (nuclear), and NRF2 (total) in indicated groups were measured. (d) Log2 (fold change) value of *Pfkfb1-4* genes from RNA-seq. (e and f) mRNA and protein levels of PFKFB3 in indicated BMDMs and RAW264.7 cells in response to LPS (e). Further, the relative amounts of PFKFB3 in different groups were measured (f). (g) Immunoblot image (upper panel) and densitometric analysis (lower panel) revealed the effect of macrophage (BMDM and RAW264.7 cell) GIT1 on PFKFB3 expression without LPS treatment. (h) Densitometric analysis showed the relative amounts of PFKFB3 in the indicated groups were performed. (I) The relative amounts of PFKFB3 in the indicated groups were measured. (J) IVG analysis of *Pfkfb3* locus showing NRF2-binding sites (Peak1 and Peak2). Statistical analysis: one-way ANOVA with post hoc test (e-g); two-way ANOVA with post hoc test (a-c, h and i).

**Supplementary Figure 6.** (a) Representative H&E staining images in the defect region of different transplanted mice (CKO to CKO vs. GIT1^fl/fl^ to KO). Scale bar = 100 μm. (b) Infiltrated F4/80^+^ macrophages were quantified at different time points (days 3, 7, and 10) postoperatively from indicated mice. iNOS group (left panel): samples stained with anti-F4/80 and anti-iNOS; CD206 group (right panel): samples stained with anti-F4/80 and anti-CD206 (unpaired two-tailed Student’s t-test). (c) ELISA was performed to detect the concentration of IL1β in the bone defect region at different time points (days 0, 3, 7, 10, and 14) post-injury from different transplanted mice (CKO to CKO vs. GIT1^fl/fl^ to KO) (one-way ANOVA with post hoc test).

**Supplementary Figure 1**


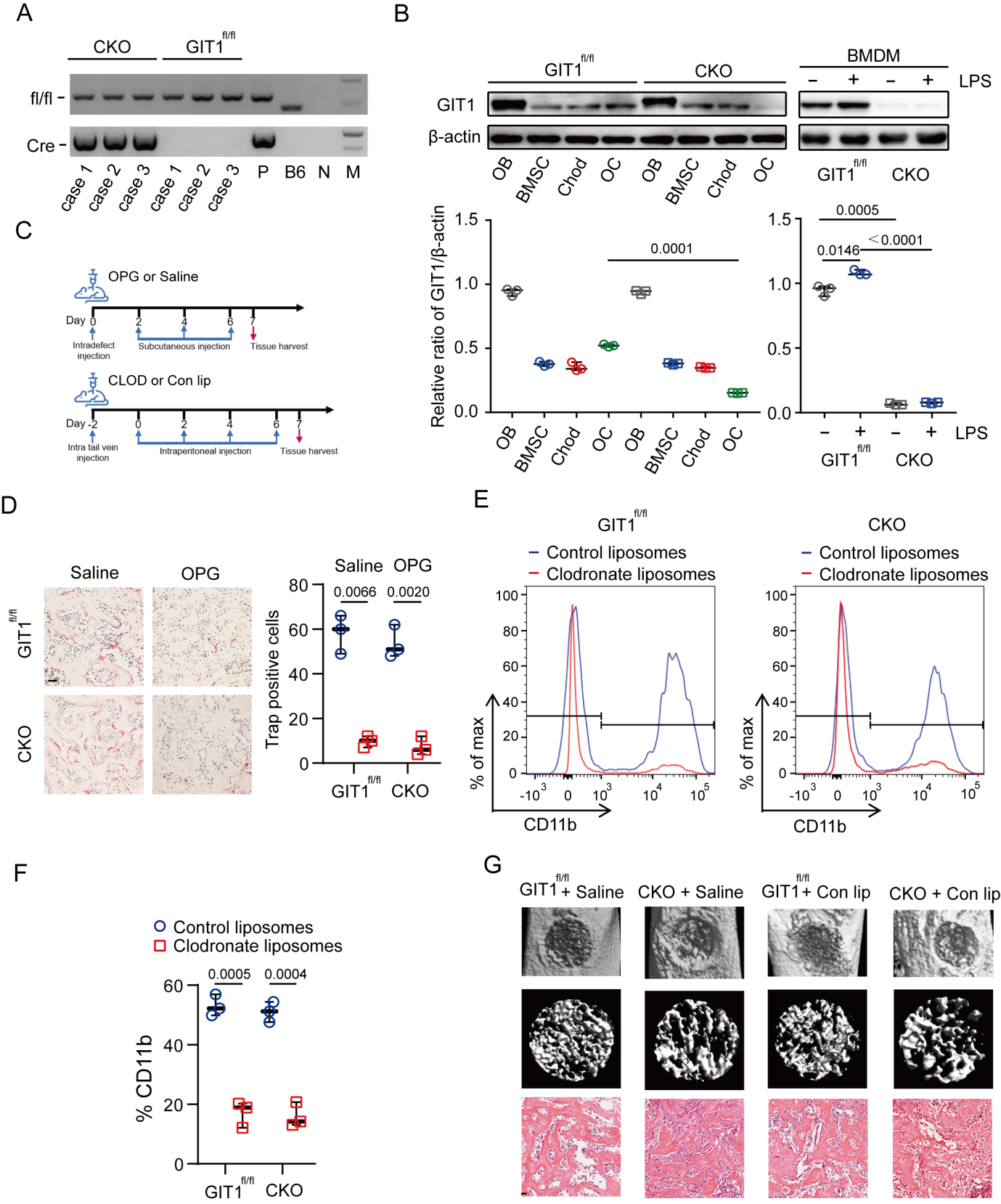


**Supplementary Figure 2**


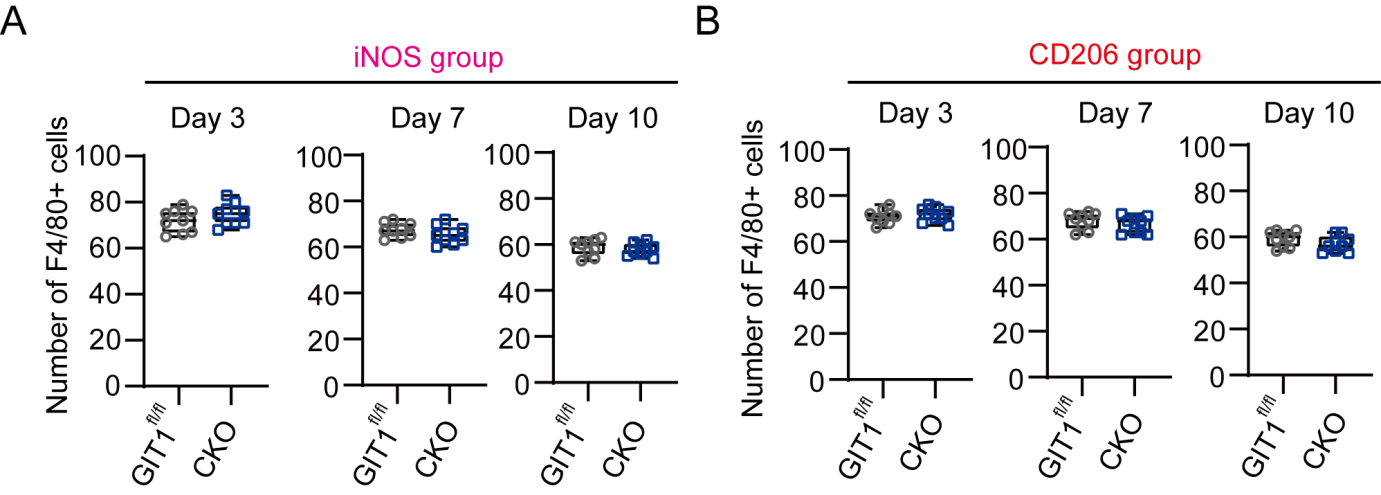


**Supplementary Figure 3**


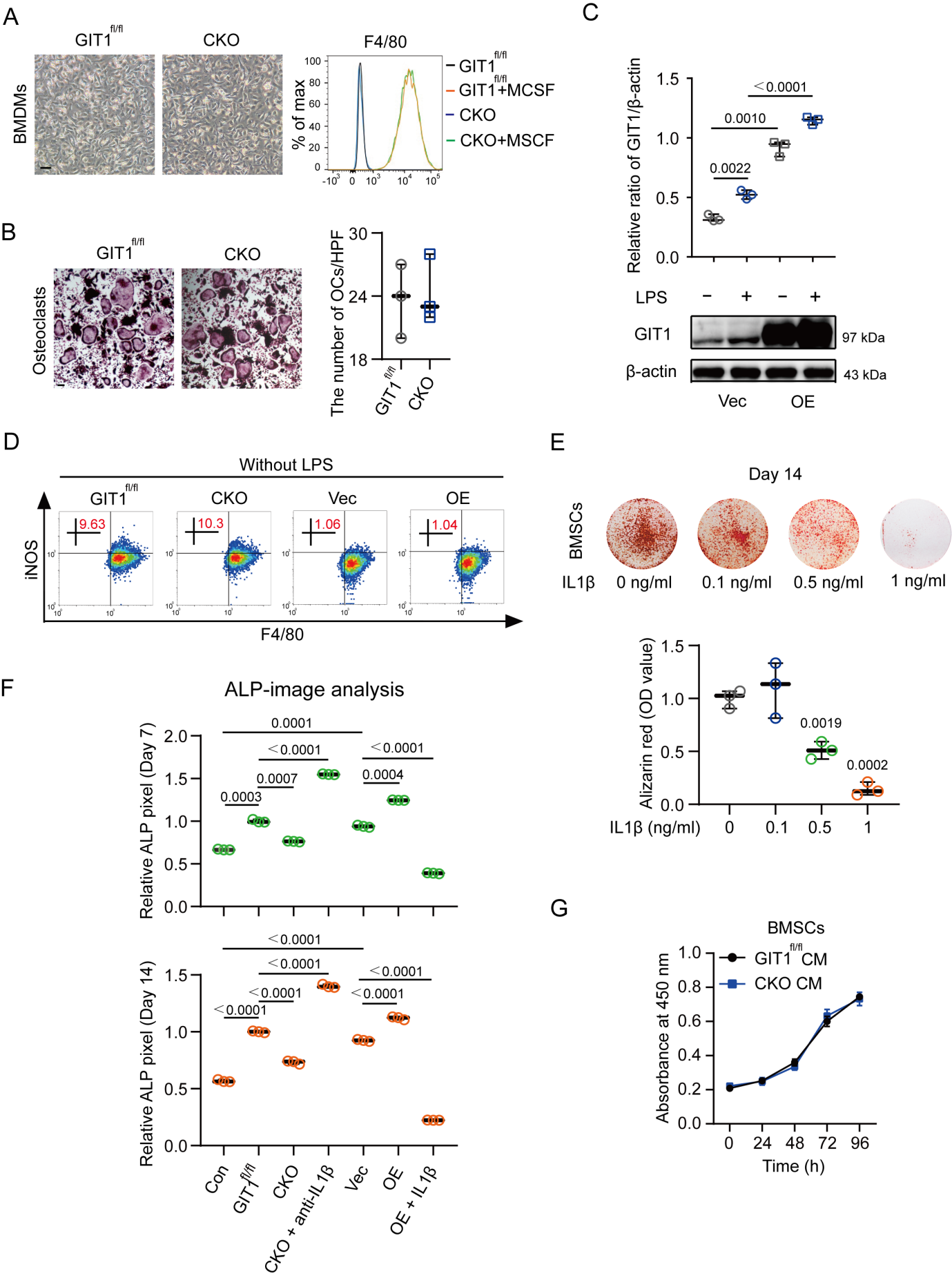


**Supplementary Figure 4**


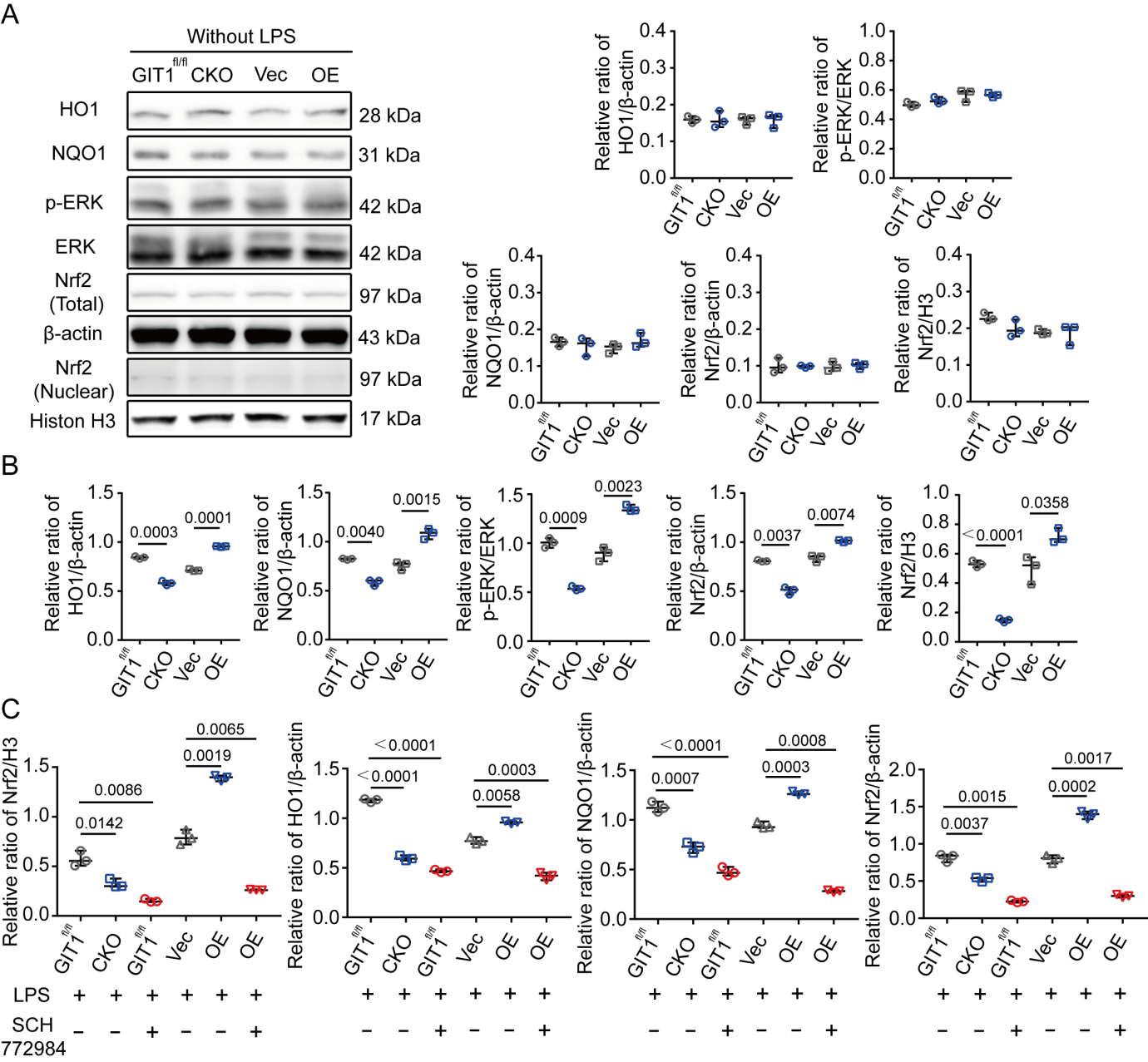


**Supplementary Figure 5**


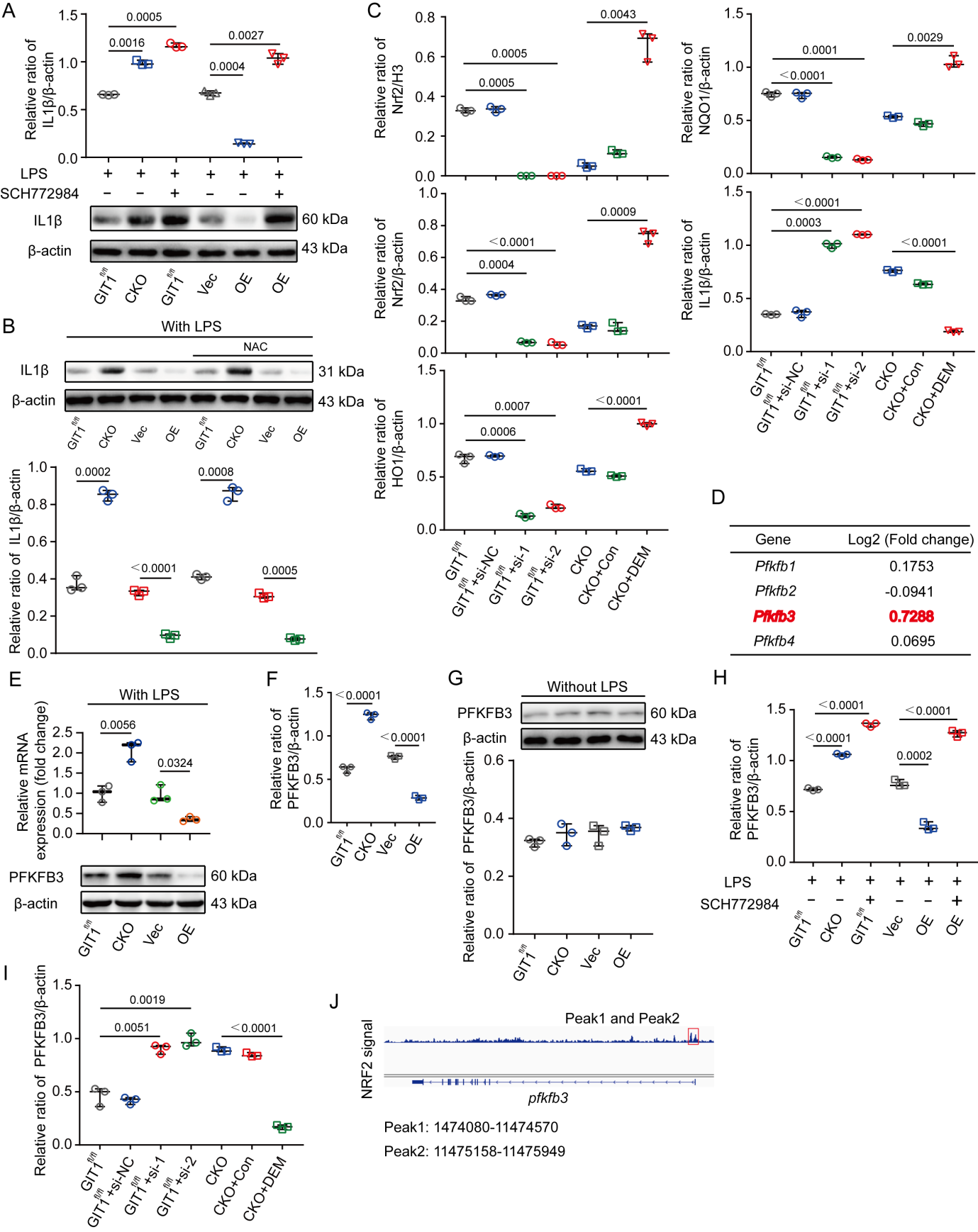


**Supplementary Figure 6**


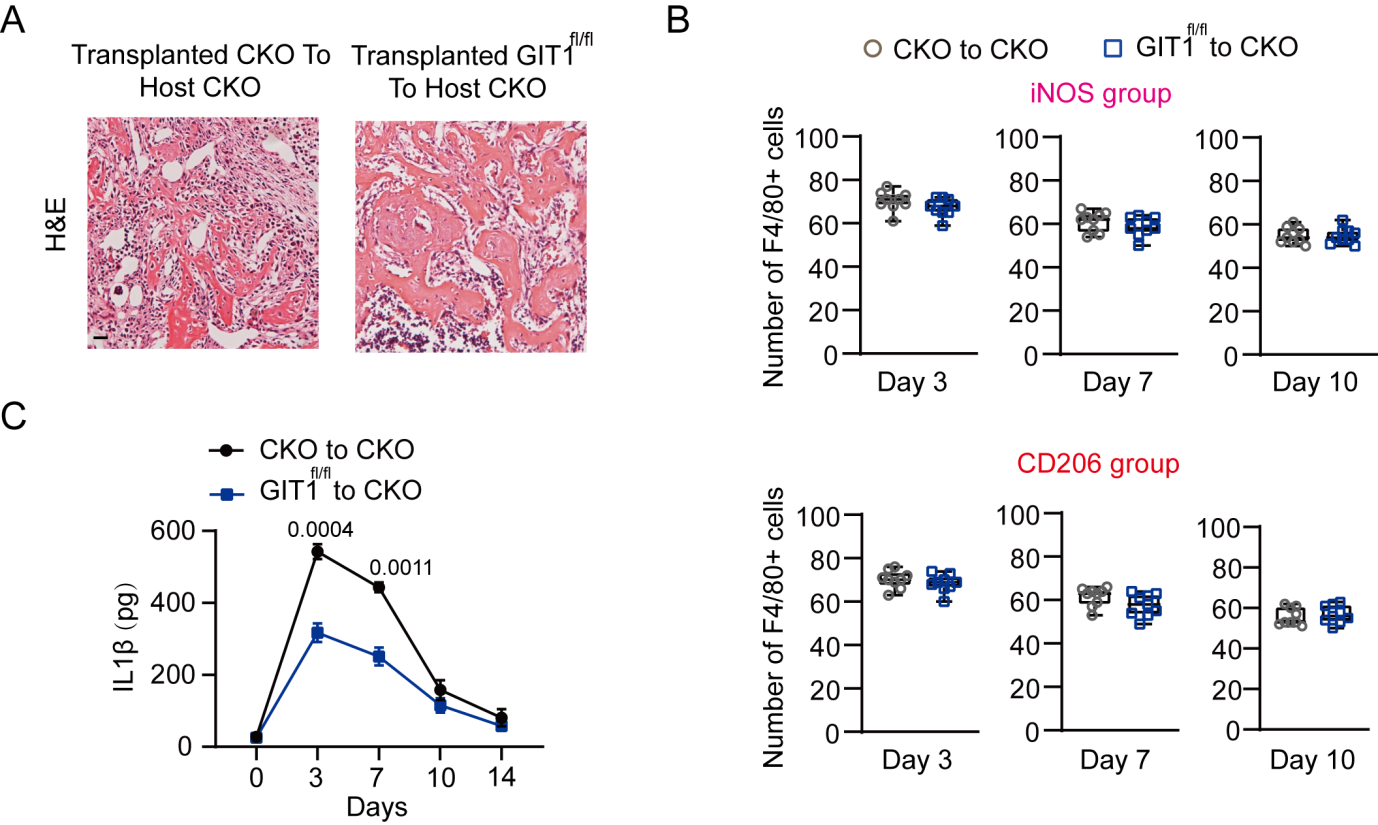

Supplement: Supplementary file 1 — Supplemental Fig. S1. (A) GIT1fl/fl; Lyz2‐Cre+ offspring were crossed with each other, genomic DNA was extracted from tails, and genotyping was detected by PCR. GIT1fl/fl Lyz2‐Cre mice were considered myeloid‐specific GIT1 CKO mice and were named CKO. P = positive control; B6 = genomic DNA from B6 mice (negative control); N = no‐template control. (B) Expression patterns of GIT1 in several kinds of primary cells from GIT1fl/fl and GIT1 CKO groups were identified using Western blotting. Densitometric analysis showed the relative amounts of GIT1 in the indicated groups (lower panel). OB = osteoblast; BMSC = bone marrow stromal cell; Chod = chondrocyte; OC = osteoclast; BMDM = bone marrow‐derived macrophage. (Left panel: one‐way ANOVA with post hoc test; right panel: two‐way ANOVA with post hoc test). (C) Schematic representing the experimental design of OPG, CLOD, and the relative control regent injection regimen, and tissue harvest. OPG = osteoprotegerin; CLOD = clodronate liposomes; Con lip = control liposome. (D) Number of TRAP+ cells in the injury region using TRAP staining revealed a significant decrease in TRAP+ cells after OPG treatment in both GIT1fl/fl and GIT1 CKO groups. Scale bar = 100 μm. (E) After treatment with clodronate liposomes or control liposomes, representative flow cytometry images indicated the percentage of CD11b+ cells in peripheral blood in GIT1fl/fl and GIT1 CKO mice. (F) Quantification of the percentage of CD11b+ cells in the indicated groups suggested that the majority of monocytes/macrophages are depleted with CLOD (one‐way ANOVA with post hoc test). (G) Representative images of micro‐CT reconstruction of injured tibias, mineralized callus, and H&E staining in the defect area of indicated groups. Scale bar = 100 μm. Supplemental Fig. S2. (A, B) Infiltrated F4/80+ macrophages were determined at indicated time points postoperatively from GIT1fl/fl and GIT1 CKO mice using IF staining. iNOS group (A) revealed the tissues stained with anti‐F4/ [file JBMR-35-2015-s001.docx]
